# Ray-Trace Modeling to Characterize Efficiency of Unconventional Luminescent Solar Concentrator Geometries

**DOI:** 10.1021/acsaom.3c00074

**Published:** 2023-05-11

**Authors:** Shomik Verma, Daniel J Farrell, Rachel C. Evans

**Affiliations:** † Department of Materials Science and Metallurgy, University of Cambridge, 27 Charles Babbage Rd, Cambridge CB3 0FS, U.K.; ‡ Exciton Labs, Copley Hill Business Park, Cambridge Road, Babraham, Cambridge CB22 3GN, U.K.

**Keywords:** luminescent solar concentrators, ray tracing, Monte Carlo, 3D-printing, open-source software

## Abstract

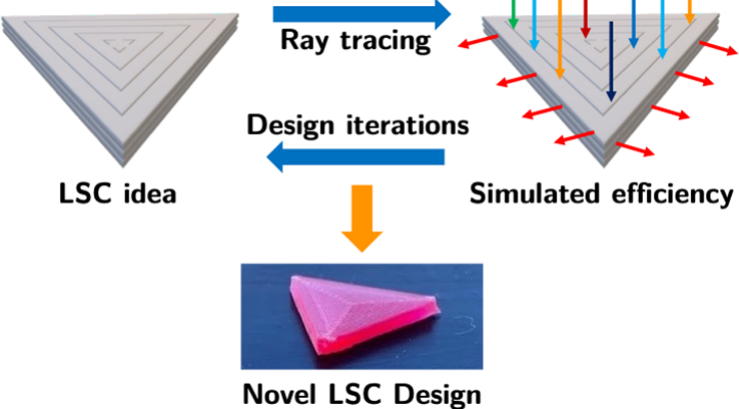

Luminescent solar concentrators (LSCs) are a promising technology to help integrate solar cells into the built environment, as they are colorful, semitransparent, and can collect diffuse light. While LSCs have traditionally been cuboidal, in recent years, a variety of unconventional geometries have arisen, for example, circular, curved, polygonal, wedged, and leaf-shaped designs. These new designs can help reduce optical losses, facilitate incorporation into the built environment, or unlock new applications. However, as fabrication of complex geometries can be time- and resource-intensive, the ability to simulate the expected LSC performance prior to production would be highly advantageous. While a variety of software exists to model LSCs, it either cannot be applied to unconventional geometries, is not open-source, or is not tractable for most users. Therefore, here we introduce a significant upgrade of the widely used Monte Carlo ray-trace software pvtrace to include: (i) the capability to characterize unconventional geometries and improved relevance to standard measurement configurations; (ii) increased computational efficiency; and (iii) a graphical user interface (GUI) for ease-of-use. We first test these new features against data from the literature as well as experimental results from in-house fabricated LSCs, with agreement within 1% obtained for the simulated versus measured external photon efficiency. We then demonstrate the broad applicability of pvtrace by simulating 20 different unconventional geometries, including a variety of different shapes and manufacturing techniques. We show that pvtrace can be used to predict the optical efficiency of 3D-printed devices. The more versatile and accessible computational workflow afforded by our new features, coupled with 3D-printed prototypes, will enable rapid screening of more intricate LSC architectures, while reducing experimental waste. Our goal is that this accelerates sustainability-driven design in the LSC field, leading to higher optical efficiency or increased utility.

## Introduction

**Figure 1:**
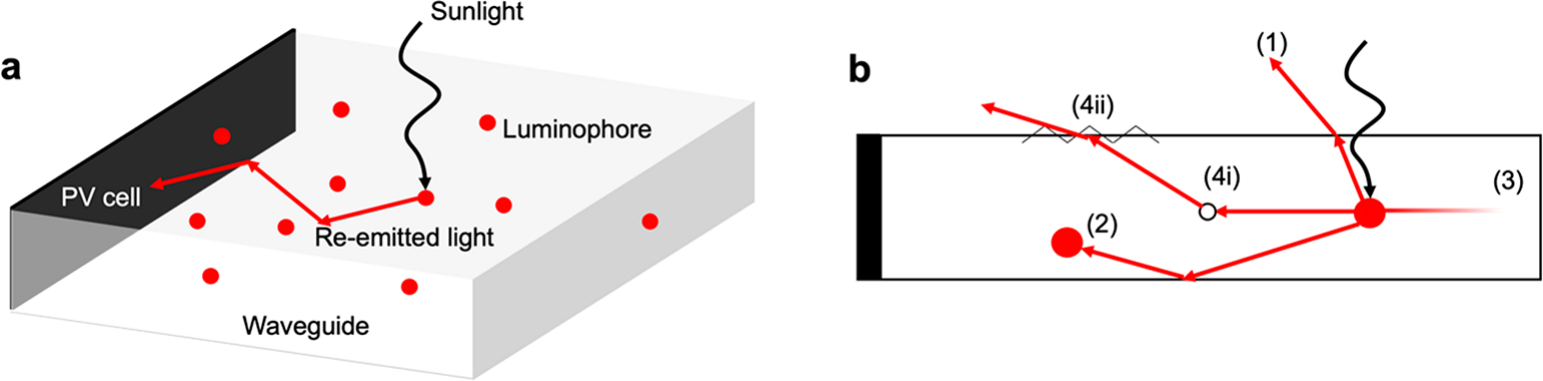
Operation and losses in an LSC. (a) Schematic of an LSC showing incident sunlight being absorbed and re-emitted by a luminophore, after which it is transported by total internal reflection to the waveguide edge, where a PV cell is installed. (b) Schematic depicting the various optical loss pathways of an LSC. (1) Escape cone losses, (2) reabsorption, (3) waveguide absorption, (4i) waveguide scattering, and (4ii) surface scattering.

Luminescent solar concentrators (LSCs) are light-harvesting components fabricated from a transparent waveguide slab that is doped or coated with a luminescent species (luminophores).^1^ They collect solar radiation over a large surface area, upon which it is spectrally converted via photoluminescence (PL) and redirected to the edges of the device where photovoltaic (PV) cells can be mounted (Figure 1a).^2^ LSCs can be used in low intensity and diffuse lighting and offer many advantages for manufacturing and design, such as being lightweight and having low-cost form factors as well as customizable colors.^3^ These features have led to highly innovative proposals for new applications of LSCs beyond standard PV, which have recently been highlighted.^4,5^

While LSCs are a useful complementary technology to PV, they suffer from optical loss pathways that can limit the amount of incident sunlight that eventually reaches the PV cells, as illustrated in Figure 1b.^6^ Although it was initially believed that LSC geometry had little effect on the efficiency,^7^ more recently, alternative LSC designs, beyond rectangular slabs, have been explored to help reduce these optical losses and also facilitate incorporation into everyday items.^3^ Some examples include cylinders^8^ and circles,^9^ stacked LSCs,^10^ curved LSCs,^11^ polygons,^12^ wedges,^13^ leaf tiles,^14^ and mosaic tiles.^15^ However, the fabrication of new LSC designs can be time- and resource-intensive. Typical manufacturing techniques either require specific casting molds to be made or use wasteful subtractive techniques starting from larger stock material. Robust simulation methods capable of quantitatively predicting the performance of nonstandard LSC design before fabrication are therefore urgently needed to accelerate screening beyond a trial-and-error approach.

While mathematical models evaluating standard optical events have accompanied experimental results since LSCs were first proposed in the 1970s,^16,17^ in recent years, Monte Carlo ray tracing has emerged as the preferred approach to simulate LSC performance.^18^ In this approach, individual rays (photons) are traced through the LSC geometry, with probabilities being assigned to different events such as reflection, transmission, absorption, emission, and scattering.^18^ The various probabilities are sampled with Monte Carlo techniques, and when sufficiently large numbers of rays (>10 000) are used, an accurate representation of LSC performance can be achieved. It is therefore straightforward to apply the same model architecture to various LSC geometries, offering a significant advantage over optical^7,19^ or thermodynamic^20,21^ models of performance.

Early Monte Carlo ray tracing studies focused on rectangular LSCs using organic luminophores and included basic optical events such as reflection/refraction at LSC faces, waveguide background absorption, and absorption/re-emission by the luminophore.^22^ Further iterations by other groups have seen consideration of more complex optical effects such polarization and dye alignment,^23^ waveguide and polymer host scattering,^24^ or alternative luminophores such as quantum dots.^25^ Due to the simplicity of this method, several groups have developed their own in-house Monte Carlo ray-tracing software for rectangular LSCs.^26−28^ More recently, there has been an emergence of ray-tracing programs to simulate unconventional LSC geometries. For example, Kennedy et al. built a ray tracing program to evaluate device performance of rectangular, triangular, hexagonal, and circular LSCs,^12^ Hughes et al. compared wedge-shaped to planar LSCs using their own software,^13^ while Zhang et al. developed a 3D ray-tracing program to simulate LSCs with bottom-facing PV cells.^29^

A common feature of all these studies is that they required custom-made ray-trace codes to address a specific characteristic. Some studies opt to use commercial ray-tracing software, such as LightTools,^30,31^ GoldSim Pro,^32,33^ or OptisWorks.^34,35^ However, these commercial codes can be expensive and inaccessible for the wider community. The lack of a versatile, open-source, user-friendly software capable of modeling the performance of different LSC designs is thus still a considerable barrier to progress in the field. Fortunately, there have been some recent efforts to democratize ray-tracing software. Zhang et al. reported an open-source Monte Carlo ray-tracing software with a graphical user interface (GUI) to model conventional rectangular LSCs, thus increasing accessibility to those unfamiliar with programming.^36^ Smith et al. published a versatile open-source ray-tracing software capable of simulating a variety of 3D geometries, conducting validation studies on planar and wedge-shaped LSCs with scattering phosphor films.^37^ However, the most widely used open-source ray-tracing software for LSCs is pvtrace,^38,39^ which has previously been used to model rectangular^18,38,40^ and cylindrical/fiber LSCs.^41,42^ It has also been used to study more unconventional geometries, such as luminescent solution-filled cuvettes,^43^ luminescent photomicroreactors,^44^ LSCs with aligned nanorods,^45^ the electric mondrian,^46^ and PV leaf roof tiles.^47^ pvtrace has been validated against other LSC models, both thermodynamic^40^ and ray-tracing,^18,21,27^ as well as against experimental results.^18,40,48^

Despite the versatility of pvtrace, it still contains some inherent limitations. First, the code is relatively inflexible in terms of photon output emission counting, as only the position of each exit photon is known and not which face the photon is exiting from. Further, the code is relatively slow, as it is serialized to model one photon at a time, and simulations of geometries with many curved surfaces can be inefficient to run. Finally, although the code is open-source and well-documented, it still requires a level of programing knowledge in to obtain useful results.

Herein, we report three upgrades to pvtrace that both extend its modeling capability and improve its accessibility to the wider LSC community, namely: (i) we advance the output ray-counting mechanism to better match standard experimental measurement conditions^49,50^ and enable simulation of exotic geometries; (ii) we add parallelization options to reduce simulation time, and (iii) we implement a graphical user interface to improve usability. Importantly, we demonstrate the versatility of these upgrades using three case studies, which span both different LSC geometries and fabrication methods, in which the simulated LSC efficiency is directly benchmarked against the measured efficiency of fabricated devices. Notably, we demonstrate that huge potential for pvtrace used in conjunction with 3D-printing, to simulate and then fabricate prototypes of bespoke LSC designs, with excellent agreement between the simulated and measured LSC efficiency.

## Methodology

### Computational Details

pvtrace is an open-source Monte Carlo ray-tracing software used to model luminescent materials—in particular LSCs—in which rays are created and followed from incidence to exit to generate a statistical distribution of ray outcomes. The original pvtrace code,^51^ written in Python and developed by Daniel Farrell,^38^ features a top-down architecture to compartmentalize material and optical properties. A schematic of the code architecture is shown in Figure S1. The ray-tracing occurs in a *scene*, which is a data structure consisting of nodes that can be designated as *geometry* or *light*. Within each *geometry*, general material properties such as refractive index can be defined, or more specific features such as surface characteristics, absorption/scattering coefficients for the waveguide, and absorption and emission spectra and/or emission direction for luminophores can be added. Each *light* source can be similarly tuned to have a specific direction, divergence, and wavelength spectrum. pvtrace conducts ray-tracing by tracking each generated ray through the defined geometries while displaying a 3D visualization of the simulation. Once ray tracing is complete, the predicted external photon efficiency (η_ext_) of the LSC for the defined parameters is calculated as
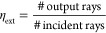
1where the output rays are defined as rays hitting an arbitrary collector surface and the incident rays are those hitting the top surface of the LSC, as defined by the *light* node. While the original code enabled ray tracing of both built-in (*Box*, *Cylinder*, and *Sphere*) and user-defined (through the import of STL files) LSC architectures, calculation of the external photon efficiency was limited to rectangular systems due to the methodology used to count input and output rays. In this study, we modify pvtrace to introduce novel, and importantly, flexible ray-counting mechanisms, which enable more complete characterization of unconventional device geometries (see Results section for details). The input parameters used to model the efficiency of different LSC designs can be found in the Supporting Information (see Section S6).

### LSC Fabrication and Characterization

**Figure 2:**
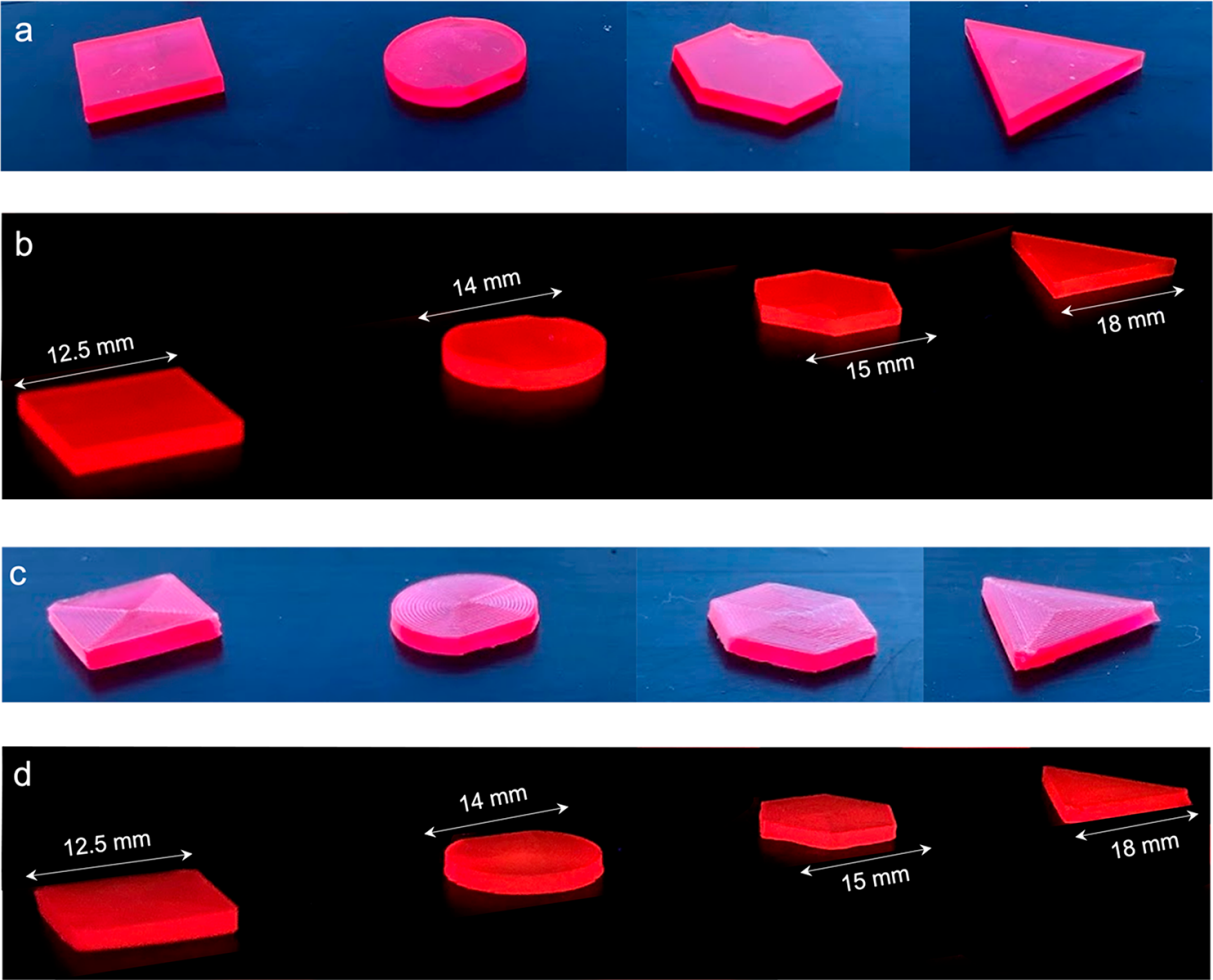
(a,b) Bulk LSCs formed by laser cutting PMMA slabs (1.6 mm thickness) doped with LR305. From left to right, the shapes are box (square), circle (cut), hexagon, and (equilateral) triangle. Each shape has (approximately) the same top surface area. Shapes are shown under (a) daylight and (b) UV illumination. (c,d) 3D-printed LSCs formed with a PMMA filament doped with LR305 (0.01 wt %), under daylight and UV illumination, respectively. Shape geometries are the same as bulk parts. Further dimensions are available in Table S6.

To verify the code modifications made to pvtrace, simulated η_ext_ values were benchmarked against experimentally determined η_ext_ values for a series of LSC geometries, namely a square, cut circle, hexagon, and triangle (Figure 2). These geometries were chosen as they had at least one flat edge, so that measurement of the edge emission was possible. Two different manufacturing methods were used: (i) laser cutting from bulk cast polymer slabs and (ii) 3D-printing via fused-deposition modeling (FDM).

Laser cut samples were prepared from cast poly(methyl methacrylate) (PMMA) slabs (thickness = 1.6 mm) doped with Lumogen Red 305 (LR305) at ∼100 ppm, which were a kind gift from Prof. Michael Debije (Eindhoven University of Technology). Analogous geometries were printed using a fused-deposition modeling (FDM) 3D printer (Prusa MK3S i3), which was chosen for ease-of-use. Custom LR305-PMMA filament was prepared in-house from a physical mixture of PMMA pellets (Sigma-Aldrich, MW = 120 kDa) with LR305 powder (100 ppm, BASF), which was extruded using a screw extruder (Noztek Pro). LSCs were printed using a layer height of 0.05 mm, line width of 0.4 mm, infill of 100%, and concentric printing pattern. Further details of the extrusion and 3D-printing parameters, along with the dimensions and geometric gain of each design are available in the Supporting Information (see Tables S4 and S7).

The optical performance of LSCs was measured using a previously reported experimental setup (see Figure S2).^52^ In brief, the LSC was illuminated with a solar simulator (Class ABB, AM1.5G, Abet Technologies) equipped with an AM1.5G filter. The LSC was supported on a bespoke 3D-printed sample holder, with one edge directly aligned with the port of an INS125 integrating sphere (225–1400 nm, International Light Technologies) to capture emitted photons. A black card was placed below the LSC, and black tape was used on edges not being measured to minimize back- and side-scattering events. The distance of the solar simulator above the sample was calibrated such that 1 sun (1000 ± 10 W/m^2^) of illumination on the top surface of the LSC was attained. The emission spectrum from the single edge of the LSC was collected by a calibrated spectrometer (SpectriLight ILT 950), which was connected to the integrating sphere via a fiber optic cable. This process was repeated for each edge of the LSC. Conversion of the measured spectra to number of emitted photons (through integration), followed by summation of all measured edges, yields the required total photon output (cf. number of output rays in eq 1).

## Results and Discussion

### Expansion of Efficiency Determination to User-Defined Geometries

In principle, the previous version of pvtrace (v2.1.2)^53^ could model diverse user-defined LSC architectures through the import of STL files. However, in practice, quantitative modeling to obtain the external photon efficiency was only possible if the user had knowledge of Python programming language and reasonable skill. This is because the in-built method used to determine the number of rays emitted from the LSC was tuned to rectangular box LSCs; namely, the program obtains the *x*- and *y*- values of each exiting ray and compares these with the dimensions of the box in its local coordinate system. If the position of the ray matches the box dimensions, then the ray is labeled as an output ray; otherwise, it is ignored. The external photon efficiency was then calculated by dividing the number of output rays by the number of incident rays. Unfortunately, this approach cannot be easily extended to geometries such as circles, hexagons, or triangles, which would require complex equations to determine which *x*- and *y*-coordinates belong to which edge. Additionally, pvtrace v2.1.2 only has two incident light patterns available—rectangular and circular^53^—which are insufficient to model additional geometries.

To extend the capability of pvtrace to include quantitative modeling of the efficiency for user-defined LSC architectures, in this work we upgrade the output ray counting mechanism. Instead of comparing the exit position of each ray to predefined *x*/*y*-coordinates, we now compare the exit surface of each ray to predefined collector surfaces, where PV cells may be placed on the LSC. In this approach, three distinct methods may be used to identify collector or noncollector surfaces. In each case, the collector surface is nominally on the edge of the LSC, orthogonal to the light-harvesting surface. The conglomeration of all pvtrace modifications is referred to as pvtrace v2.1.sv and is freely available on GitHub,^54^ and these changes will be integrated with pvtrace in future versions.

**Figure 3:**
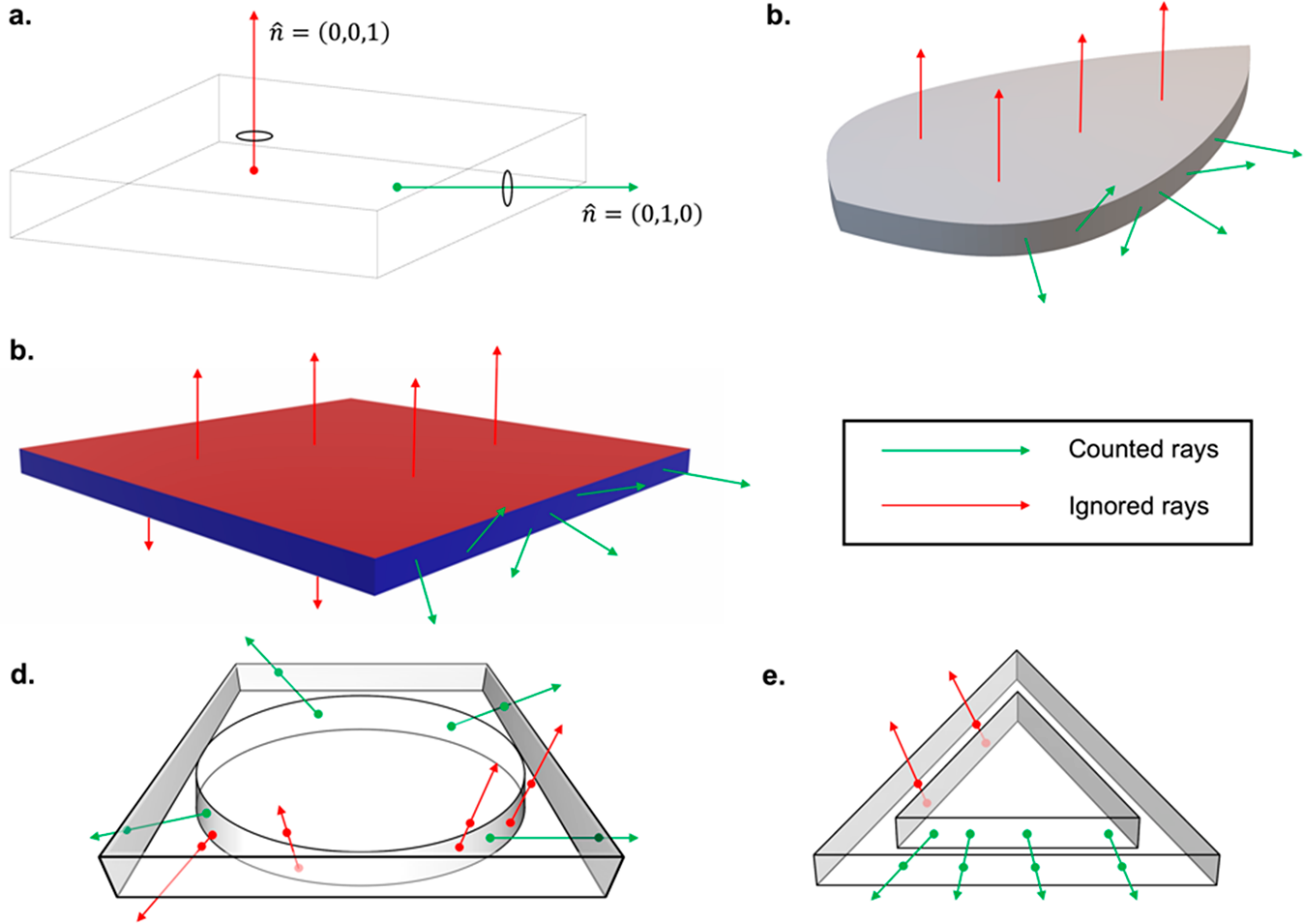
Different output ray counting mechanisms used to predict the external photon efficiency of user-defined LSC geometries: green arrows denote counted rays, while red arrows corresponds to ignored rays. (a) and (b) illustrate the surface normal approach for rectangular and leaf geometries. Only exit rays that are orthogonal to the incident surface are counted. In the case of the leaf structure, although the directions of the side exit rays are random, the surface normal rays are consistent. (c) illustrates the color edge-emission counting approach, showing the edges of the LSC colored as blue and the incident surface as red. This method offers improved manual control over the identification of edge-emission surfaces. (d) depicts an example of the enclosing box method, in which all rays exiting from the edges of the enclosing box surrounding an LSC (here circular) are counted as output rays. A subset of this approach is the enclosing shape method shown in (e), in which a scaled-up version of the geometry is used to enclose the actual LSC.

First, is labeling using *surface normals*. This method calculates the normal of the surface hit by the exiting ray. If this normal is in the desired output direction, the ray is counted. This method is most useful for modeling LSCs that have PV cells installed on their edges, but any desired output direction can be chosen. Figure 3a shows an example of the surface normal approach applied to a rectangular box. Rays exiting from the top will have a surface normal of (0,0,1), while rays exiting from the right side will have a surface normal of (0,1,0). If PV cells are placed on the right side of the LSC, it would suffice to count all rays with exit surface normal (0,1,0) as output rays. Figure 3b shows the more complex geometry of a “leaf” LSC. In this case, we assume flexible solar cells are placed along the edges of the leaf; thus, any exit ray with surface normal with *z*-value equal to 0 counts as an output ray. This method is advantageous, since we no longer need to create a complex formula to describe the coordinates of the leaf edges against which exiting rays are compared.

The second method for surface labeling involves using *colors* in the input CAD files used to specify the LSC geometry. While STL files do not contain colors in their metadata, other file formats (e.g., DAE files) do. Figure 3c shows an example of the *color* method, where the LSC edges are set as blue, while the incident surface is red. In this approach, only rays exiting from a blue edge would count as output rays. Although the *surface normal* method is convenient as it automatically detects edge surfaces, the *colors* method offers more flexibility in LSC design, as the user can manually define surfaces where PV cells could be installed.

The final approach—the *enclosing box* method—was implemented to better mimic the experimental approach commonly used (as in this study) to determine the external photon efficiency, in which the LSC edge is placed at the input to an integrating sphere connected to a spectrometer to measure the intensity and wavelength of output light. To imitate this in simulation, instead of counting all rays exiting from the edges of the device, the device is first enclosed in a box, and all rays exiting from the edges of the enclosing box are counted as output rays. An example of this for a circle LSC is shown in Figure 3d. A subset of the *enclosing box* method is the *enclosing shape* method, shown in Figure 3e for a triangle LSC. Instead of enclosing the LSC in a box, the LSC is enclosed in a scaled-up version of itself. This helps to replicate experimental setups where the total edge emission is measured by sequentially pointing individual edges at an integrating sphere and summing the edge emissions to obtain the final output.

**Figure 4:**
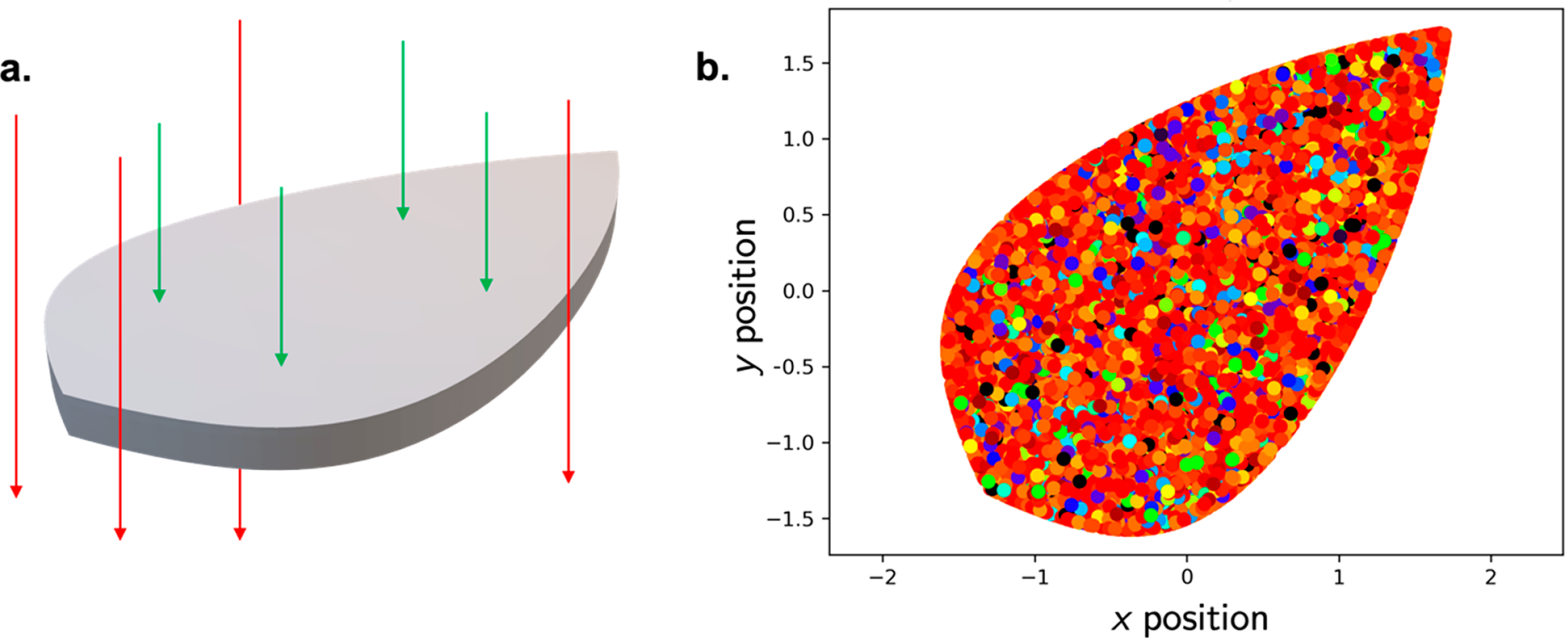
(a) Demonstration of the modified light mask implementation. Green arrows correspond to counted light rays, while red arrows are ignored. (b) Results of pvtrace simulation showing modified mask ignoring all rays missing the object, which shows the *x*/*y* position of all entrance and exit rays of a leaf LSC with LR305 luminophore. Colors represent wavelengths of incident and re-emitted rays.

The final modification included in pvtrace v2.1.sv eliminates the geometrical requirements of the incident light mask. In this new approach, rays are generated in an *x*- by *y*-shaped rectangle, which is just big enough to cover the part, but if a ray misses the part, it is ignored. We can thus generate a cleaner light input consisting only of rays that are incident on the object. We note that any interaction with the geometry is recorded, so transmission losses are still included, meaning the optical efficiency calculated by pvtrace corresponds to external photon efficiency (η_ext_), as defined in eq 1, instead of the internal optical efficiency (η_int_), which would only consider absorbed rays in its efficiency calculation (see eq S2 in Section S3). We further note that if a ray completely misses a part, it is replaced to ensure the total number of rays incident on the part matches the user input. Figure 4 shows the result of this modification for a leaf LSC, plotting the *x*- and *y*- positions and wavelengths of entrance and exit rays.

### Improving Computation Time with Parallelization

The simulation of more complex geometries that could have many more surfaces than rectangular LSCs will lead to increased computational time. Fortunately, the ray-tracing problem is considered embarrassingly parallel, since each ray is independent and does not interact with other rays.^55^ For this reason, the simulation can be split across multiple computing cores to speed up computational time. This is especially useful with access to a supercomputer with dozens of cores. For example, if a user wanted to simulate 10 000 rays, a supercomputer with 10 cores could assign 1000 rays to each core, vastly improving computation time. In this work, we use either the multiprocessing module^56^ or the Ray module,^57^ depending on whether pvtrace v2.1.sv is running on a personal machine or a supercomputer, respectively, to parallelize its processes. Simulations were performed on a hypothetical cylindrical (6 cm diameter, 0.32 cm height) LSC using a rectangular mask of incident light and the *surface normal* method to demonstrate the effect of parallelization on computational time.

An easy way to reduce computation time is to parallelize pvtrace just using the cores available on a PC/Mac, since most laptops and desktops have multiple cores. The multiprocessing package^56^ creates a pool of all available cores and distributes tasks among them in the most efficient way possible, which typically means the rays are evenly distributed among the cores. The advantage of this approach is immediately apparent: implementation of the multiprocessing pool of the 2 cores available on the 2017 MacBook Pro results in a 50% decrease in computation time (for either 1 000 or 10 000 rays) compared to serialized (nonparallelized) simulations using only 1 computing core (see Figure S3a). While a ∼100 s run time is not prohibitively high, this will increase significantly for more complex parts, such as STLs with multiple surfaces, motivating the need for parallelization. It is also possible to run the code on a computing cluster. Clusters typically have several nodes, and each node has multiple CPUs or cores. Figure S3b shows the results of running 10 000 rays in parallelized pvtrace on the Cambridge computing cluster,^58^ running on a single node with a multiprocessing pool of 1 through 16 cores, with a power curve fit plotted. The equation of the curve fit is shown, and the exponent of *x* close to −1 proves the nature of the power fit.

Since the multiprocessing module only supports single-node computation, to access additional nodes on the supercomputer we instead used the Ray module^57^ for distributed computation. Ray allows programmers to use the same syntax as multiprocessing, still creating a pool of cores, but can handle communication between nodes. Using *n* nodes should reduce completion time by *n* times compared to single-node computation. Figure S3c shows the computation time using a Ray pool, 100 000 rays, and an increasing number of cores. While the trend is initially linear, indicating a power curve fit, the relationship breaks down as the number of cores employed is increased, as computation time reaches a minimum. This is likely due to the overhead created by the Ray package having to initialize pvtrace on each node, establishing a minimum runtime. However, parallelization clearly results in a massive reduction in the runtime of pvtrace. This new script thus increases the quality of simulations while saving time in users’ workflows.

### Improving User Experience with a Graphical User Interface (GUI)

**Figure 5:**
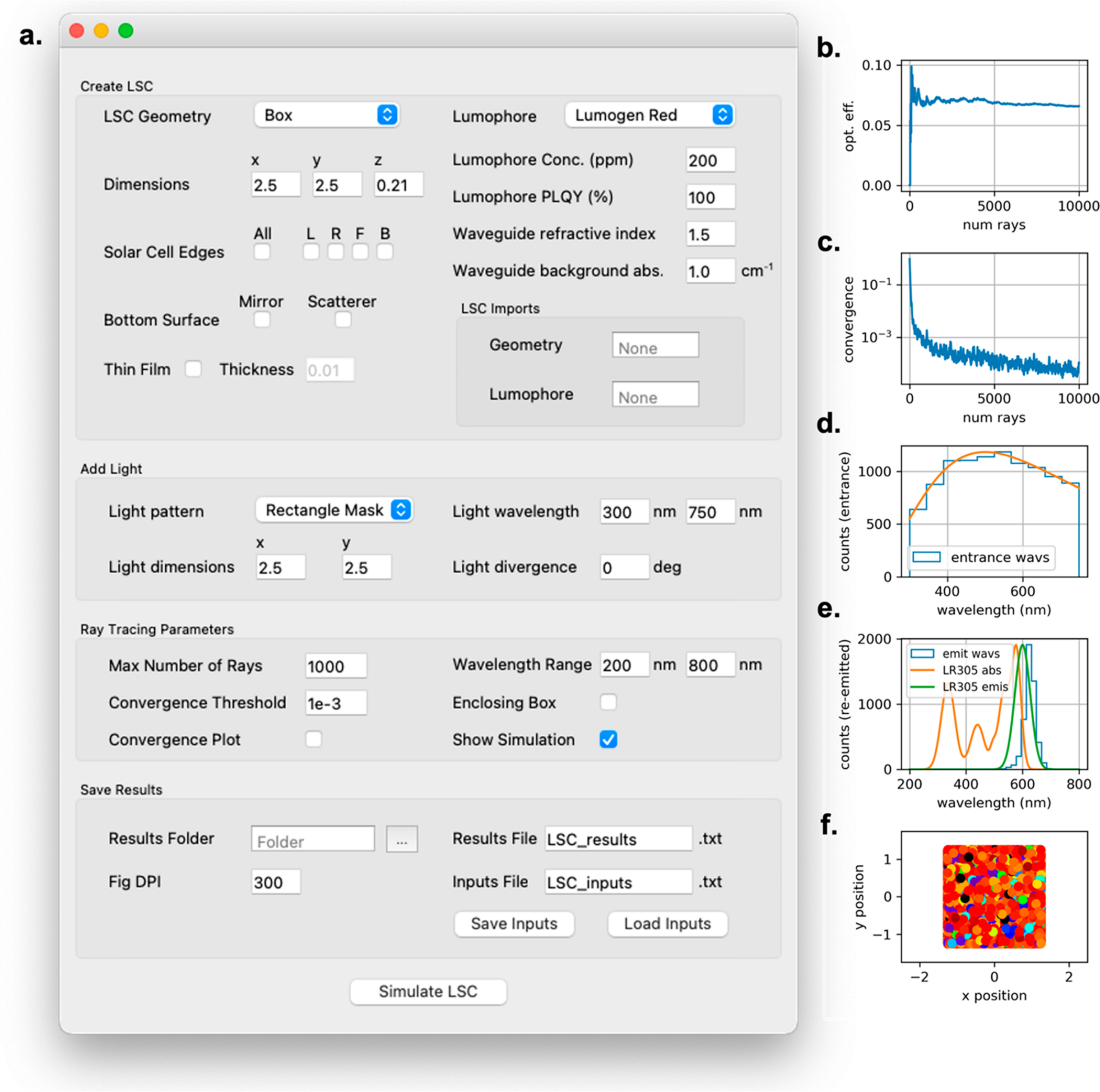
(a) pvtrace GUI showing all capabilities. Basic steps include creating an LSC, adding light, setting ray-tracing parameters, and saving results. Clicking “Simulate LSC” using the default values will create a Box LSC with 2.5 × 2.5 × 0.2 cm dimensions, 200 ppm LR305 in PMMA (1.0 cm^–1^ background absorption), with a solar spectrum of light incident on top. The pvtrace GUI generates five output plots to help the user assess the quality of the simulation: (b) The external photon efficiency (opt. eff) as the simulation generates more rays. (c) The convergence of the efficiency toward a final value. (d) The wavelength of generated entrance rays versus the input incident spectrum. (e) The wavelength of exit rays versus the absorption/emission spectra of the luminophore, and (f) the *x*- and *y*-positions and wavelengths (represented by colors) entrance and exit rays.

Experimental research groups working on LSCs may not have extensive experience with programming. As such, there is a steep learning curve to using the original pvtrace program, which may have limited its widespread use to date. The final addition in this work is the inclusion of a GUI with the aim of making pvtrace more accessible to a diverse potential user base. Figure 5a shows a screenshot of the GUI developed in this work and the various inputs required for the simulation. A typical workflow for using the GUI is described below.

First, the user must create an LSC. The geometry of the LSC can be a Box, Cylinder, Sphere, or users can also import their own STL files. The LSC dimensions (automatically updated for imported STL files) must be provided as well as any additions to the LSC including the attachment of PV cells at the edge(s), which would result in refractive index matching and lower exit surface reflections, or the placement of a scattering/reflective bottom surface, which could help recycle photons. It is also possible to select a thin-film geometry here. In this case, a thin layer (of user-specified thickness) of luminophore-doped material is placed on top of a bulk waveguide slab, and the user inputs a luminophore concentration for only the thin film. The efficiency is then calculated as normal. For both bulk and thin-film geometries, the luminophore must be selected—either Lumogen F Red 305 (LR305), which is built in, or the absorption and emission spectra of another luminophore can be imported as a CSV file. The absorption spectra of the luminophores are in the form of an attenuation coefficient, which can be multiplied by the luminophore concentration to get the absorption coefficient (see Section S5 for further details). Finally, the specific values of key optical properties can be entered, such as the concentration (ppm) and photoluminescence quantum yield (PLQY, %) of the luminophore and the refractive index and background absorption (cm^–1^) of the waveguide. Note that the luminophore concentration in the thin film should be higher than in the bulk part to achieve similar performance.

Next, the pattern/shape of the incident light is selected. Rectangular masks are the default, though the in-built circular mask or point source may also be selected. The dimensions of the light source will automatically update to match the size of the LSC. The wavelength range and divergence of the light source must be input by the user.

Third, the ray-tracing parameters must be set. The maximum number of rays for the simulation and the convergence threshold (details described below) are used to determine how long the simulation will run. The wavelength range is used to calculate the luminophore spectrum from a polynomial fit of the input absorption and emission spectra and to ensure the absorption/emission is zero beyond the bounds of the luminophore. The *surface normal* method of efficiency measurement is used by default, but the *enclosing box* method can be selected if an alternative method for ray counting is desired. Checking either the “convergence plot” or “show simulation” boxes will display either as requested.

Finally, the GUI makes it possible to easily save results. Users can choose a folder location and file name to save the results, as well the resolution of the output figures (see below). It is also possible to save the input data in a file or load an input file for ease of repeating simulations.

Using the GUI, the simulation will return the total external photon efficiency (shortened to optical efficiency (opt. eff.) in the program), as well as the efficiency at each edge of the device (of course, for alternative geometries, the efficiencies at the four cardinal directions have little meaning). In addition, as shown in Figure 5b–f, the GUI also outputs five data plots to help the user assess the quality of the simulation. Figure 5b shows the optical efficiency as the program runs and generates additional rays. This is useful for the user to know if this parameter changes significantly while the program is running or if it is generally stable. Figure 5c shows the convergence plot of the efficiency. Convergence is defined as the difference between each new efficiency value (as rays are added to the simulation) and the average of all previous values. If the convergence value reaches below the threshold (default 10^–3^, user-configurable), the simulation is said to converge, and the simulation automatically stops. Figure 5d shows the distribution of entrance wavelengths superimposed on the input spectrum used, to prove that they match. Figure 5e shows the distribution of exit wavelengths along with the absorption and emission spectra of the luminophore. This is useful to visualize any reabsorption losses caused by the dye and to ensure that the optical properties of the luminophore were incorporated accurately. Finally, Figure 5f shows the *x*- and *y*-positions and wavelengths of the entrance and exit rays, which is a useful qualitative visualization of efficiency (e.g., note the red dots on the device edges) and to ensure the object was correctly detected.

### Demonstrating Applications of Upgraded pvtrace

Older versions of pvtrace (<v2.0) have been compared to experimental data previously^18,40,41^ and showed strong correlation between the analytical and measured LSC efficiency.^41^*Ad hoc* simulations of LSCs using pvtrace v2.1.2^53^ and pvtrace v2.1.sv, developed in this work,^54^ show completely equivalent results, indicating the new additions to the code did not impact analytical results. For a more rigorous comparison, we now present three case studies demonstrating the application of pvtrace v2.1.sv to (1) square LSCs, (2) unconventional LSC geometries, and (3) 3D-printed LSCs.

#### Case Study 1: Square LSCs Prepared by Casting

Cast LSCs based on the red-emitting dye LR305 embedded in a PMMA slab in rectangular geometry dominated the early scientific literature in the field^1^ and are commonly used as a standard against which to benchmark the performance of new materials combinations.^59^ LR305 typically exhibits a high photoluminescence quantum yield (PLQY > 90%)^60^ in the solid state, is dispersible in diverse solvents and matrices, and shows good photostability.^61^ However, its small Stokes shift leads to significant overlap of its absorption and emission spectra (see Figure S4), meaning reabsorption losses are common in LR305-based LSCs.^62^ PMMA satisfies most of the requirements required to minimize waveguide losses, namely a refractive index of ∼1.49, good optical clarity, and high transmittance across a large portion of the solar spectrum.^1^

**Figure 6:**
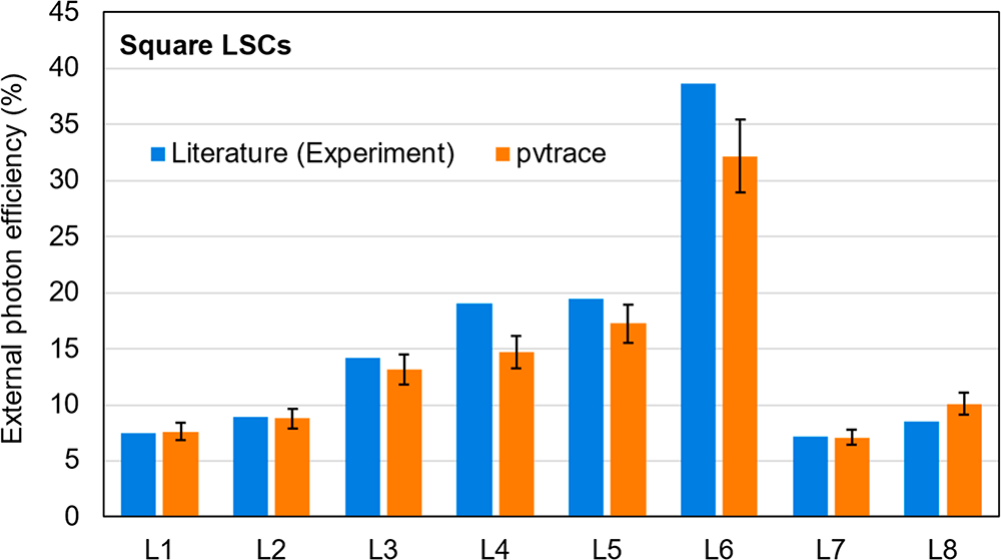
Comparison between simulated (pvtrace v2.1.sv) and measured external photon efficiency from literature reports of bulk square LSCs doped with LR305 in PMMA from different laboratories. 10 000 rays were used to simulate efficiency. To obtain error bars, the rays are split into 10 groups of 1000, and the standard deviation was taken, which can be considered a worst-case estimate as convergence was likely not achieved after 1000 rays. Full simulation inputs and numerical efficiency results are available in Tables S2 and S3. L1, L2 from Zettl et al.;^59^ L3, L4 from Desmet et al.;^10^ L5, L6 from Bose et al.;^48^ L8, L9 from Debije et al.^65^

Key examples from the literature reporting the efficiency of square LR305-PMMA LSCs were selected to compare the ability of pvtrace v2.1.sv to predict the external photon efficiency. Care was taken to exclude any examples that used engineering approaches to enhance the LSC performance, e.g., the use of reflective or scattering layers on the bottom or exposed edges. The important parameters from each study were LSC dimensions, LR305 concentration, refractive index, waveguide parasitic absorption, light source spectrum, and edge-emission measurement methodology (see Table S2 for values). The parameters were input into pvtrace v2.1.sv and simulated with 10 000 rays. If the experimental study used PV cells to measure efficiency, the *surface normal* method was used; otherwise, the *enclosing box* method was used. The results of the comparison are shown in Figure 6.

Overall, the comparisons indicate pvtrace v2.1.sv retains its accuracy against a wide variety of square LSCs, produced and measured in different laboratories. The predicted external photon efficiency is within 1% for most examples and within 7% for all examples. However, several difficulties were encountered in making these simulations, which could explain the deviation between the simulated and experimental efficiencies. The PLQY of many organic dyes, including LR305, decreases with increased concentration due to aggregation,^63^ but this effect was not included in the simulation, which may impact the results for LSCs using high LR305 concentration. The method of determining the edge-emission output also varied between studies; for example, while most studies used an integrating sphere to collect emitted photons (see Methodology section), Zettl et al.^59^ used a fiber optic cable (20 μm diameter) placed at the center of one of the edges of the LSC. Since the photon output has been shown to fluctuate along the edge length,^64^ this approach may have artificially inflated the photon output in this study. Regardless, while fine-tuning the model with better input data could have improved results, pvtrace v2.1.sv shows strong agreement with previously reported experimental studies.

#### Case Study 2: Unconventional LSC Geometries Prepared by Casting

Many unconventional geometries have been reported previously (e.g., cylinders^8^ and circles,^9^ stacked LSCs,^10^ curved LSCs,^11^ polygons,^12^ wedges,^13^ leaf tiles,^14^ and mosaic tiles^15^), but the lack of standardization in fabrication and measurement makes comparison with simulation challenging—as observed in the previous case study. To eliminate this variable, we fabricated our own unconventional LSC geometries from cast PMMA slabs (thickness = 1.6 mm) doped with LR305 (100 ppm), which were laser cut to the desired shape (square, hexagon, triangle, and circle bulk parts, see Figure 2(a,c) for dimensions). The external photon efficiency for each geometry was measured using the integrating sphere method (see Experimental and Section S3 for details).

**Figure 7:**
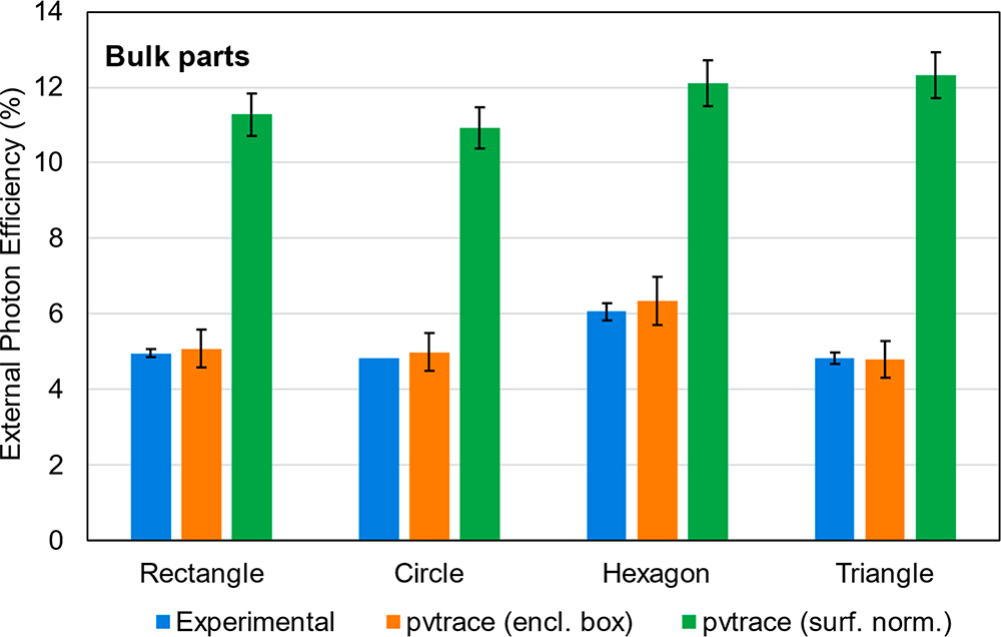
Comparison between simulated (pvtrace v2.1.sv) and measured external photon efficiency for unconventional LSC geometries (bulk parts). LSCs were fabricated from cast PMMA doped with LR305 (100 ppm), which was laser cut to the desired shape. Error bars are standard deviations of efficiencies measured at each edge of the LSC (experimental) or efficiencies obtained from splitting the 10 000 rays into 10 groups of 1000 (simulated), which can be considered a worst-case estimate as convergence was likely not achieved after 1000 rays. Note the enclosing shape method was used to best mimic the integrating sphere experimental setup. Full inputs and numerical efficiency values are available in Tables S4 and S5.

The experimental and simulated optical efficiencies obtained using both the *surface normal* and *enclosing shape* methods in pvtrace v2.1.sv are shown in Figure 7. For better comparison, we did not indicate PV cells were applied to the edges in the *surface normal* case, so both methods had refractive index mismatch between the sample and environment. This implies that the differences in simulated efficiency are due to the gap between the part and the collection edge, which could cause some rays to exit above or below the collecting surface of the enclosing shape. As seen, the *enclosing shape* method shows excellent agreement with experiment (Δη_ext_ = 0.15 ± 0.10%). In contrast, the *surface normal* method yields efficiencies which significantly deviate from experiment (Δη_ext_ = 6.5 ± 0.7%) and falsely predicts the trend in relative efficiency of each geometry. This discrepancy between the two methods is attributed to the experimental approach used to determine the efficiency, in which there is an air gap (∼3 mm) between the LSC edge and the input port on the integrating sphere. The *enclosing shape* method better represents this configuration, whereas the *surface normal* method is more analogous to experimental determination using PV cells directly attached to the LSC edge (no air gap). The increased η_ext_ obtained using the surface normal method may also indicate that the integrating sphere measurement underestimates the true external photon efficiency, due to the introduction of optical artifacts at the air gap (e.g., increased scattering or refraction due to the change in refractive index leading to loss of photons from the optical path).

**Figure 8:**
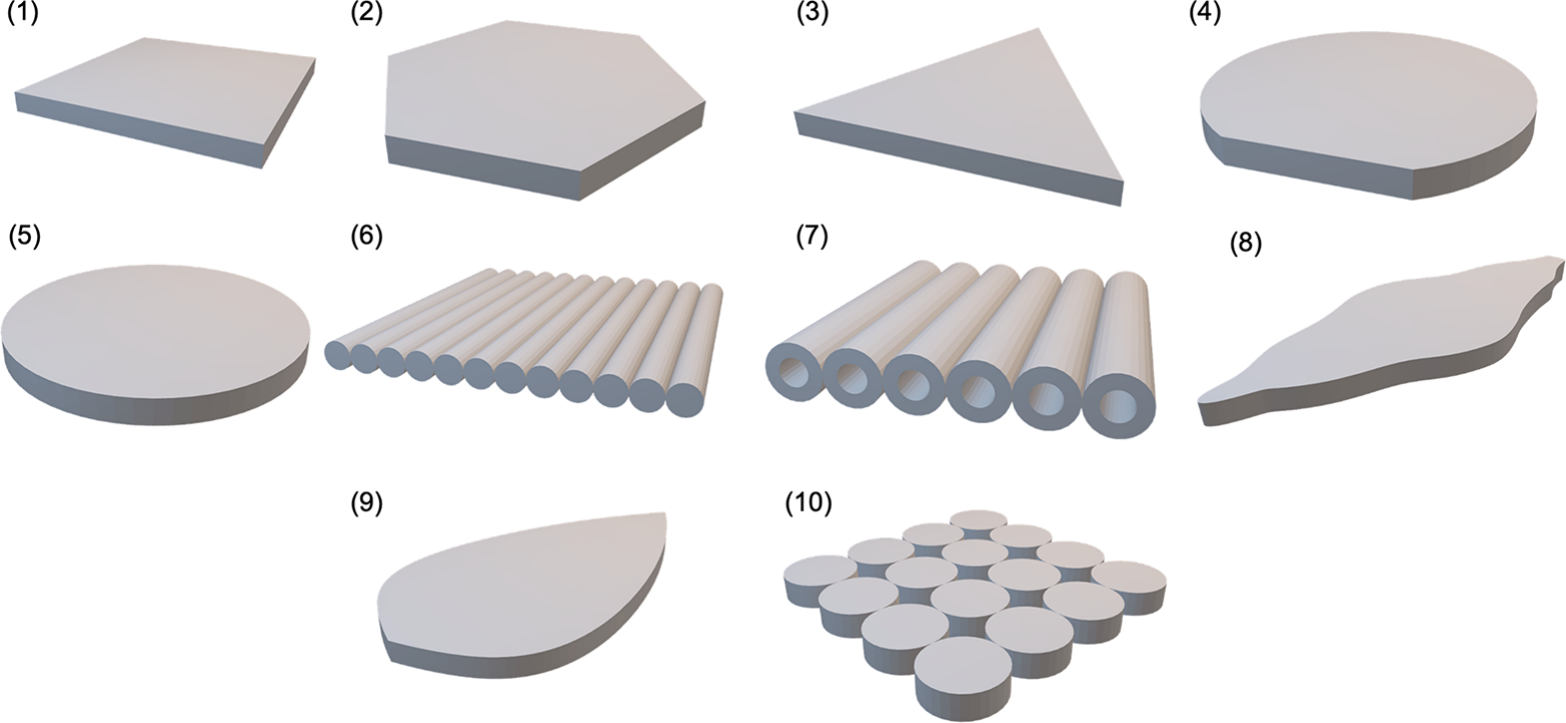
3D CAD models of bulk parts used to compare the surface normal and enclosing box methods in pvtrace v2.1.sv. (1) Square, (2) hexagonal, (3) triangular, (4) circular cut, (5) circular, (6) cylindrical array, (7) hollow cylindrical array, (8) leaf roof, (9) leaf, and (10) vertically oriented cylindrical array. Parts were standardized to all have the same top surface area (6.25 cm^2^) and thickness (0.21 cm).

Comparison of the *surface normal* and *enclosing box* methods was next extended to a wide range of potential unconventional LSC geometries. CAD files of 10 different LSC designs, some based on reported examples in the literature and some original designs, are shown in Figure 8. All designs were standardized to have a top surface area of 6.25 cm^2^ and an active absorbing depth of 0.21 cm. A conventional square LSC (1) was used for reference. Next, polygonal LSCs (hexagon (2), triangle (3)), a circle with a flat edge (4), and a regular circle (5) were used.^12^ Note that these differ from the designs in Figure 2 due to their size. Solid (6) and hollow cylindrical array (7) LSCs were also considered,^66^ along with a design LSC based on the Leaf Roof (8).^14^ Finally, original designs were used, including another leaf-like LSC (9) and a vertically oriented cylindrical array (10).

**Figure 9:**
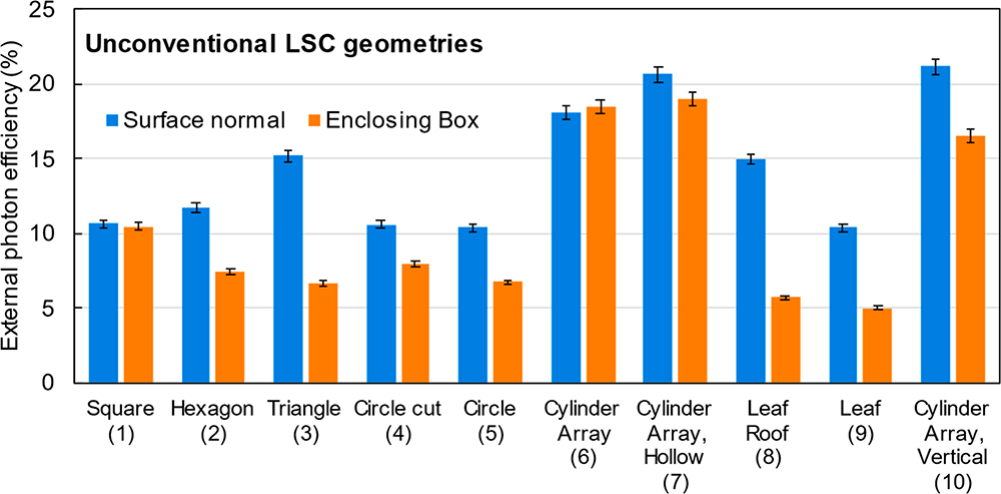
Simulated (pvtrace v2.1.sv) external photon efficiency for hypothetical unconventional LSC geometries (bulk parts). Note the use of the enclosing box method, not the enclosing shape method, to enable comparison between parts. To obtain error bars, the rays are split into 10 groups of 10 000, and the standard deviation was taken—note that this results in tighter error bars, as more rays are used than in previous results. Numbering corresponds to parts as shown in Figure 8. Full inputs and numerical efficiency values are available in Table S6.

These hypothetical LSCs were simulated with pvtrace v2.1.sv using 100 000 rays parallelized over 64 cores, using a custom light mask for each design, as described in the Methodology. Figure 9 shows the external photon efficiency calculated using both the surface normal and enclosing box methods.

The results demonstrate that pvtrace v2.1.sv can simulate a wide variety of LSC geometries. When considering just the *surface normal* method, the noncylindrical parts (2,3,8,9) produce similar to slightly higher efficiencies than the square LSC, which is in line with results of previous studies.^7,12,14^ The cylindrical parts have higher efficiencies, around 15–20%, which is also supported by experimental and simulated results.^42,67^ Note that while the parts were all standardized to have the same incident surface area, the variation in efficiency, especially for the triangle and Leaf Roof parts, could be due to different gains between parts.

When comparing the *surface normal* and *enclosing box* results, for device geometries 1, 6, and 7, the results are essentially equivalent. This is because these geometries are virtually rectangular, so adding an enclosing box around them should not change the efficiency. We note that for geometry 6, the enclosing box has a slightly higher efficiency, as fewer side surfaces were labeled as collector surfaces due to the cylindrical shape.

For all other designs, the *enclosing box* method systematically predicts a lower external photon efficiency. This is likely to be due to the increased distance that the exit rays must travel from the edge of the device to the edge of the enclosing box. Some rays may be angled such that they exit the top or bottom of the box, reducing the simulated efficiency. However, for devices 2–4, since each has a flat edge, in experiment it would be possible to align the flat edge of the device with the input port on the integrating sphere and essentially replicate the surface normal technique, providing two different ways to validate simulation. For these devices, the *enclosing shape* option instead of the *enclosing box* would be more appropriate.

#### Case Study 3: 3D-Printed LSCs

The previous case study demonstrated that pvtrace v2.1.sv can simulate the efficiency of a diverse range of hypothetical bulk LSC architectures, thus providing valuable insight into the key design criteria for high efficiency. It would be advantageous to be able to compare the simulated efficiency of these hypothetical designs with the experimental performance. However, the manufacture of intricate LSC designs from bulk LR305-PMMA is challenging. An alternative approach is to take advantage of the versatility of 3D-printing to produce rapid prototypes of these new designs.

To start, we 3D-printed LSCs in regular geometries (square, hexagon, triangle, circle) using fused-deposition modeling with custom filament of LR305-PMMA that was extruded in-house. Note that while the dimensions are identical in 3D-printed or bulk cast designs, the FDM process introduces internal surfaces into the LSC slab due to the method by which the part is manufactured (see Figure 2a vs c for comparison). We want to understand the effect that these differences in manufacturing (and thus internal slab structure) have on the LSC performance.

**Figure 10:**
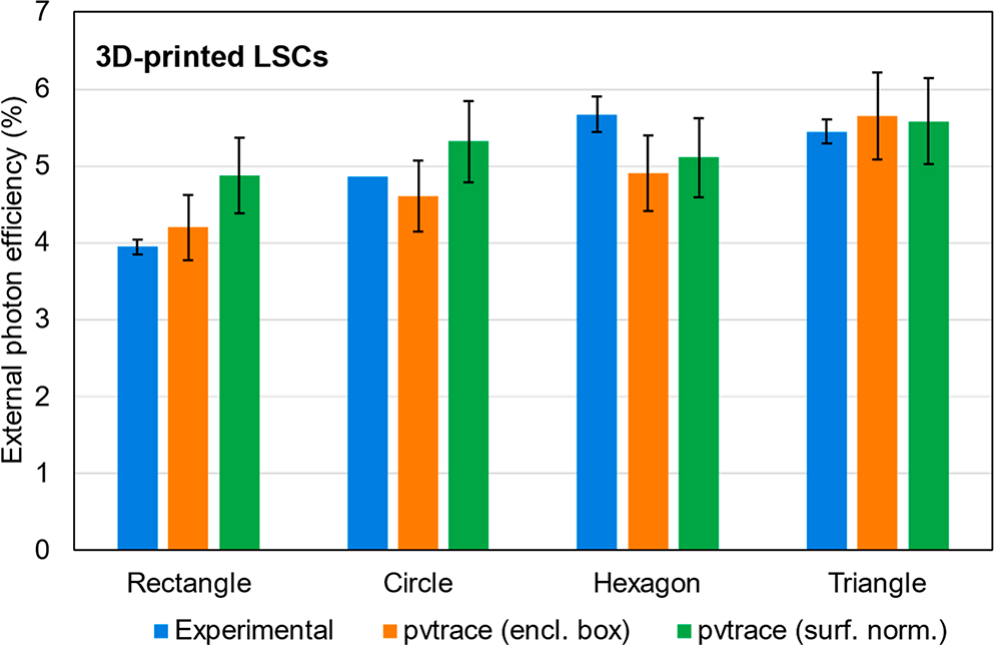
Comparison between simulated (pvtrace v2.1.sv) and measured external photon efficiency for unconventional 3D-printed LSC geometries including square, circle, hexagon, and triangle. LSCs are fabricated by 3D-printing PMMA doped with LR305. Error bars are standard deviations of efficiencies measured at each side of the LSC or efficiencies obtained from splitting the 10 000 rays into 10 groups of 1000, which can be considered a worst-case estimate as convergence was likely not achieved after 1000 rays. Full inputs and numerical efficiency values are available in Tables S7 and S8.

The external photon efficiency for each LSC was determined experimentally and simulated with pvtrace v2.1.sv with 10 000 rays using both the *surface normal* and *enclosing shape* methods, and the results are shown in Figure 10. We note that for the *enclosing shape* method, instead of scaling up the complex 3D-printed geometry, a simplified part, similar to a shape prepared by casting (no internal geometry) was used with the appropriate shape and size.

A few interesting observations can be made. First, the difference between the *surface normal* and *enclosing shape* methods is not as significant as with the bulk parts. This could be due to fewer surfaces being labeled as edge-emission surfaces in the 3D-printed parts due to the curvature inherent in the printed paths, which reduces the *surface normal* efficiency. Alternatively, it could suggest 3D-printed parts have better directionality of emission, i.e., emitted photons are more likely to be orthogonal to exit surfaces than with bulk parts. Since the FDM print process introduces a fiber-like internal structure into the bulk LSC (e.g., see Figure 2c), with each fiber separated by an air gap, it is conceivable these function as individual optical fibers that direct emission to the edges. We are currently exploring this feature in an array of 3D-printed designs to confirm this hypothesis.

We also again notice that the simulated efficiency with the *enclosing shape* method is in good agreement with the measured efficiency (*Δη*_*ext*_ = 0.4 ± 0.2%).

**Figure 11:**
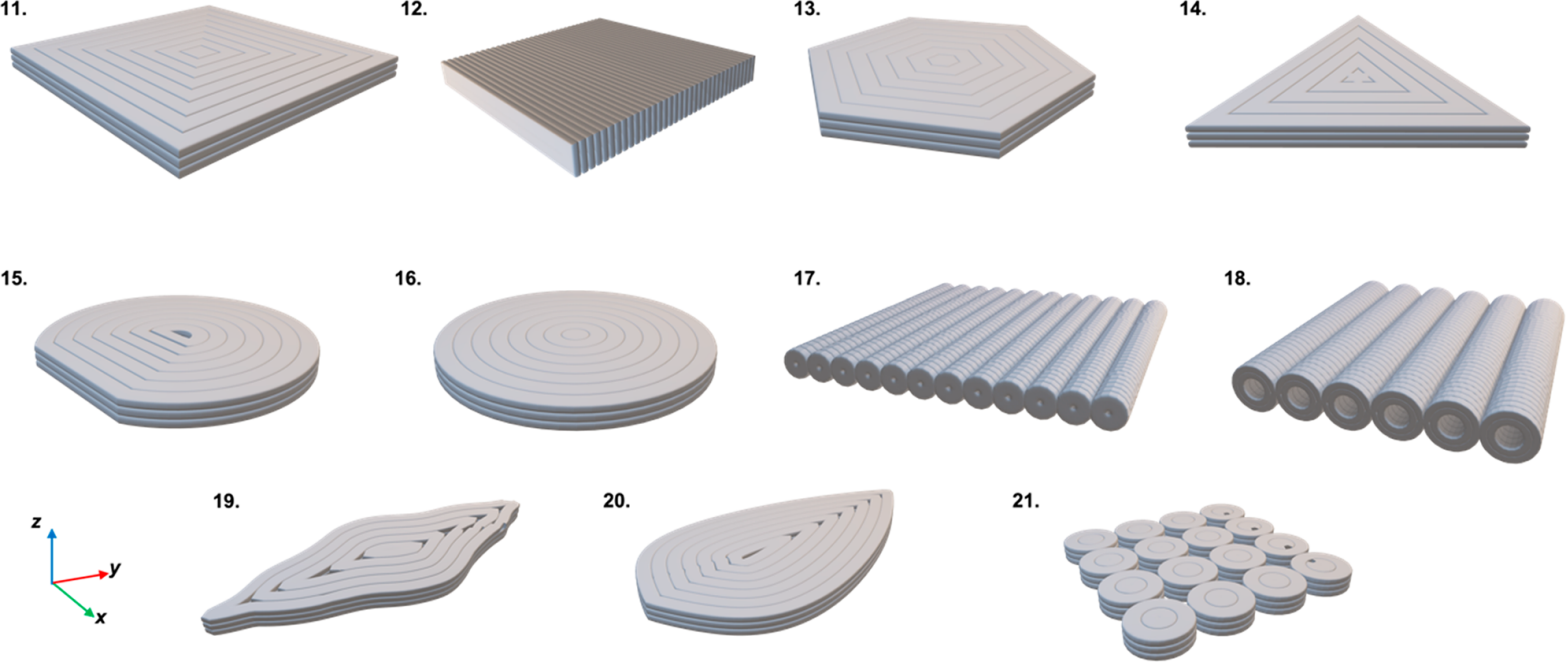
3D CAD models of concentric 3D-printed LSCs considered for simulated efficiency analysis in this study. (11) square, (12) vertically printed square, (13) hexagonal, (14) triangular, (15) circular cut, (16) circular, (17) solid cylindrical array, (18) hollow cylindrical array, (19) leaf roof, (20) leaf, and (21) vertically oriented cylindrical array. Parts were standardized to all have the same top surface area and thickness.

We next used pvtrace v2.1.sv to simulate the efficiencies of the diverse LSC designs presented in Case Study 2 but now manufactured using 3D-printing rather than bulk casting. The CAD files of the 3D-printed geometries are shown in Figure 11. Note the main difference between these parts and those in Figure 8 is the curved surfaces produced due to filament printing, which can increase scattering of light. To further demonstrate the capability of pvtrace to model 3D-printed parts, the square design was 3D-printed concentrically in two ways: (i) where the concentric paths lie on the *xy*-planes (part 11) and (ii) where the part is printed vertically such that the paths lie on the *xz* planes (part 12). The solid (part 17) and hollow (part 18) cylinder arrays were similarly vertically printed in a concentric pattern.

**Figure 12:**
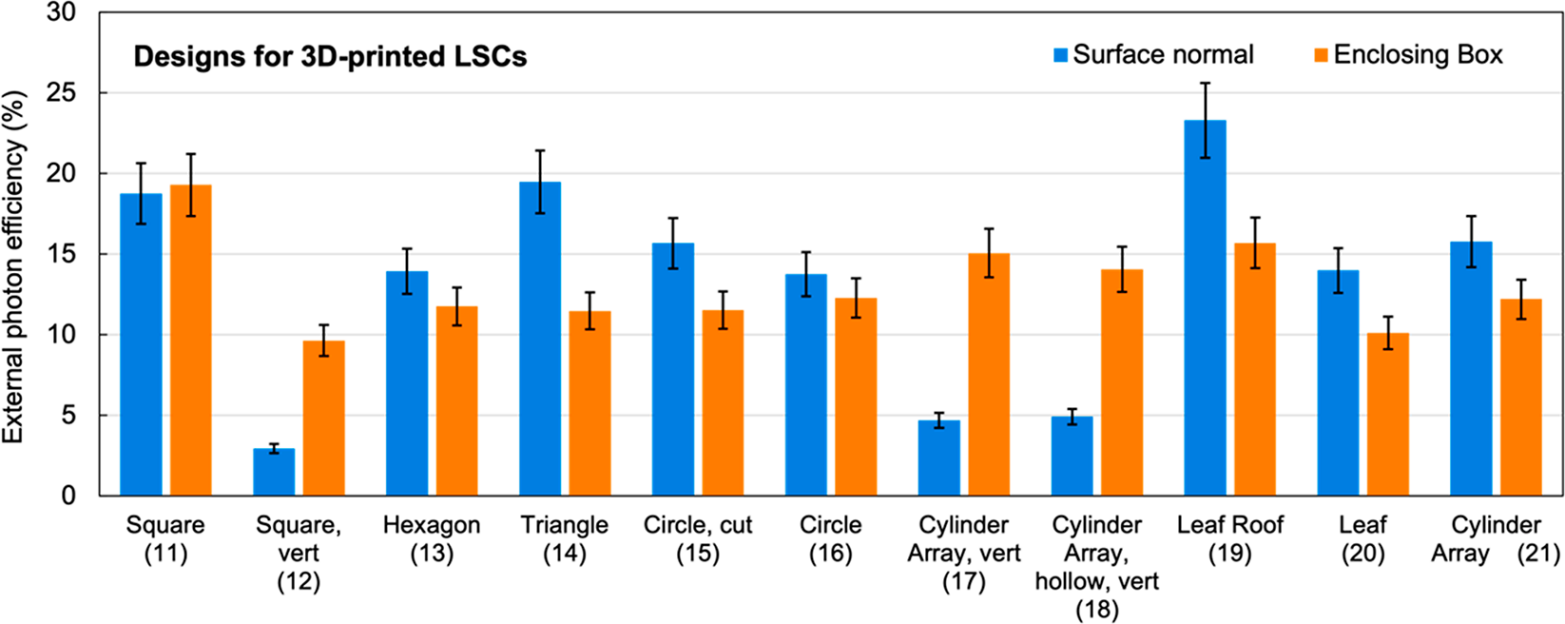
Simulated (pvtrace v2.1.sv) external photon efficiency results obtained using the surface normal and enclosing box methods for the hypothetical 3D-printed LSC architectures proposed in Figure 11. Error bars are standard deviations of efficiencies obtained from splitting the 10 000 rays into 10 groups of 1000, which can be considered a worst-case estimate as convergence was likely not achieved after 1000 rays. Full simulation inputs and numerical efficiency values are available in Table S9.

Figure 12 shows the simulated external photon efficiency calculated using both the *surface normal* and *enclosing box* methods for these hypothetical 3D-printed designs. Several key trends can be observed. First, most 3D-printed LSCs have enhanced efficiency compared to bulk devices, with a mean increase of 5 ± 2% (*surface normal*) or 6 ± 2% (*enclosing box*). There are a few potential explanations for this phenomenon. Several previous experimental and simulation studies have reported differences in optical efficiency between flat and curved LSCs, due to differences in optical path lengths and escape cone losses.^11,68,69^ Since curved surfaces are created from the printed paths, this could explain the increased efficiency. Another potential explanation is the 3D-printing paths function similarly to fiber optic cables and help guide light to the edges of the device. The result of these two effects would be increased efficiency due to improved directionality of light, as photons are guided more effectively toward the edges and are emitted more orthogonally than in bulk parts. While this is qualitatively seen in ray-tracing simulations, more rigorous experimental analysis would be needed to confirm this phenomenon.

A few designs are predicted to be less efficient when fabricated by 3D-printing versus bulk casting, namely the vertically printed parts (12, 17, 18) and the cylinder array (21). This reduced efficiency could be partially explained by the directionality arguments made above: vertically printed parts would direct more light toward the top and bottom surfaces rather than the edges, thereby leading to a decrease in the number of photons counted in the edge output. Similarly, for the cylinder array, re-emitted rays would be more likely to be emitted perpendicular to the edges and therefore enter another cylinder and potentially be reabsorbed by the luminophore or parasitically absorbed by the waveguide. Furthermore, for vertically printed parts, we see a larger reduction in efficiency in the *surface normal* calculation than the *enclosing box* calculation. This could be due to the curved surfaces introduced by the 3D-printing process, meaning fewer edge faces would satisfy the criteria of being counted by the *surface normal* method, while the *enclosing box* method is agnostic to the edge-emission surface.

Our detailed analysis has demonstrated that pvtrace v2.1.sv very accurately predicts the experimental external photon efficiency of various LSC geometries, using both bulk casting and 3D-printing fabrication techniques. Compared to literature data, the mean absolute error (MAE) was 1.98% for simulated vs measured optical efficiency; in contrast, for LSCs fabricated in this study, the MAE was remarkably just 0.25% (Figure S5). The lower MAE for LSCs prepared in-house is not unexpected, since we had direct oversight of the measurement and sample conditions. Through these comparisons to experiment, we have been able to demonstrate the utility of both the *surface normal* and *enclosing box/shape* methods. The *surface normal* method better predicts efficiencies when solar cells are used for photon measurement, due to the lack of air gap (i.e., refractive index matching) and therefore direct capture of re-emitted photons at the device edges. The *enclosing box/shape* techniques instead accurately predict efficiencies when an integrating sphere is used to characterize the device. The *enclosing shape* technique is most applicable to parts with flat edges, while the *enclosing box* technique would be better suited for curved geometries. In either case, the enclosing box/shape mitigates for the airgap between the LSC edge and the integrating sphere, which in addition to the refractive index mismatch also reduces the number of exiting photons measured.

Among the simulated devices, some cases of both under- and overestimation were observed, perhaps because there are reasons for either to occur. The model could underestimate efficiency, since it assumes the sample holder absorbs all rays, while in practice, the mask may reflect a fraction of rays. On the other hand, it could overestimate efficiency due to lack of control over surface properties and imperfections, use of a constant waveguide background absorption, and the assumption of 100% PLQY, which is unrealistic in devices based on LR305. A combination of these factors could explain why the model underestimates in certain cases while overestimating in others.

Our results have shown that pvtrace really comes into its own when modeling more exotic architectures that would be challenging to measure experimentally. The simulations reveal that, generally, for bulk parts, cylindrical designs have higher efficiency than a simple rectangular LSC. Interestingly, other modifications to the conventional LSC design show limited improvement to performance. For a 3D-printed LSC, we observed greater similarity between the *surface normal* and *enclosing box* methods, and interestingly, a higher efficiency than analogous bulk parts is predicted. In practice, however, this improvement has not yet been demonstrated experimentally for our 3D-printed LSCs, which we attribute to the low quality of our in-house extruded LR305-PMMA filament. Using pvtrace to extrapolate the potential efficiency based on a higher quality filament with reduced waveguide absorption (0.2 cm^–1^ as obtained for commercial PMMA filament vs the 5 cm^–1^ measured for our filament), a 3-fold increase in the efficiency of a square LSC is predicted, from 4.88 to 15.97%. For comparison, the corresponding bulk part has an efficiency of 11.28%. If this efficiency improvement can be validated experimentally, this may have important ramifications for future LSC designs.

## Conclusions

In summary, we have demonstrated that the upgrades we have introduced to pvtrace render it a powerful tool for pre-experimental design of novel LSC architectures with enhanced performance. By implementing different methods to detect and count the output rays, pvtrace is now able to both handle nonstandard geometries and more accurately reflect the measurement conditions when predicting the external photonic efficiency of the LSC. As nonstandard geometries increase complexity for the simulation, we have introduced parallelization to reduce computational time. The inclusion of a GUI increases the accessibility of the software to the wider LSC community and provides output plots that aid the user to monitor the progress and quality of the simulation.

We have shown that the upgrades to pvtrace developed in this work can generate key insight into the performance of new LSC designs or manufacturing techniques. This versatility is critical for the future expansion of LSCs beyond simple solar collection devices. As reviewed recently,^15,16^ LSCs have far-reaching potential for new applications such as sensing, microreactors, communication, and so on. These new applications will place different demands—and increasing complexity—on the form factors of the device. pvtrace offers a highly configurable and simple solution to simulate the optical performance of customized designs without extensive experimental effort. The direct compatibility of pvtrace (through import of STL files) with low cost, low waste 3D-printing methods for rapid screening of prototype designs is also highly attractive. We are currently exploring this potential in detail to better understand the correlation between print quality and design and simulated performance.

## Supporting Information Available

Architecture of the pvtrace code; 3D-printing methodology; experimental characterization of LSCs; results from parallelization to reduce computation time; absorption and emission spectra of Lumogen Red; input parameters and simulated efficiencies for different case studies; measured vs simulated optical efficiencies (PDF)
