# Supplementary material for: Ray-Trace Modeling to Characterize Efficiency of Unconventional Luminescent Solar Concentrator Geometries
Source: ACS Appl Opt Mater. 2023 May 11;1(5):1012–25. doi: 10.1021/acsaom.3c00074 (PMC10226161; doi:10.1021/acsaom.3c00074)
Supplement: Supplementary file 1 — ot3c00074_si_001.pdf [file ot3c00074_si_001.pdf]

Supporting Information for

Ray-Trace Modeling to Characterize Efficiency of Unconventional Luminescent Solar Concentrator Geometries

Shomik Verma[1,a], Daniel J Farrell[2], Rachel C. Evans[1]**

[1] *Department of Materials Science and Metallurgy, University of Cambridge, 27 Charles Babbage Rd, Cambridge CB3 0FS, U.K.*

[2] *Exciton Labs, Copley Hill Business Park, Cambridge Road, Babraham, Cambridge CB22 3GN, U.K.*

[a] Present address: *Department of Mechanical Engineering, Massachusetts Institute of Technology, 77 Massachusetts Avenue, Cambridge, MA 02139, USA*

* Corresponding Authors: Shomik Verma (skverma@mit.edu) and Prof. Rachel C. Evans (rce26@cam.ac.uk)

TABLE OF CONTENTS

S1 ARCHITECTURE OF THE PVTRACE CODE 2

S2 3D PRINTING OF LUMINESCENT SOLAR CONCENTRATORS (LSCS) 3

S3 EXPERIMENTAL CHARACTERIZATION OF LSCS 4

S4 PARALLELIZATION TO REDUCE COMPUTING TIME 6

S5 OPTICAL PROPERTIES OF LUMOGEN RED 7

S6 INPUT PARAMETERS AND SIMULATED OUTPUTS FROM PVTRACE 8

S7 MEASURED VERSUS SIMULATED OPTICAL EFFICIENCIES 14

S8 REFERENCES 14

S1 Architecture of the pvtrace code

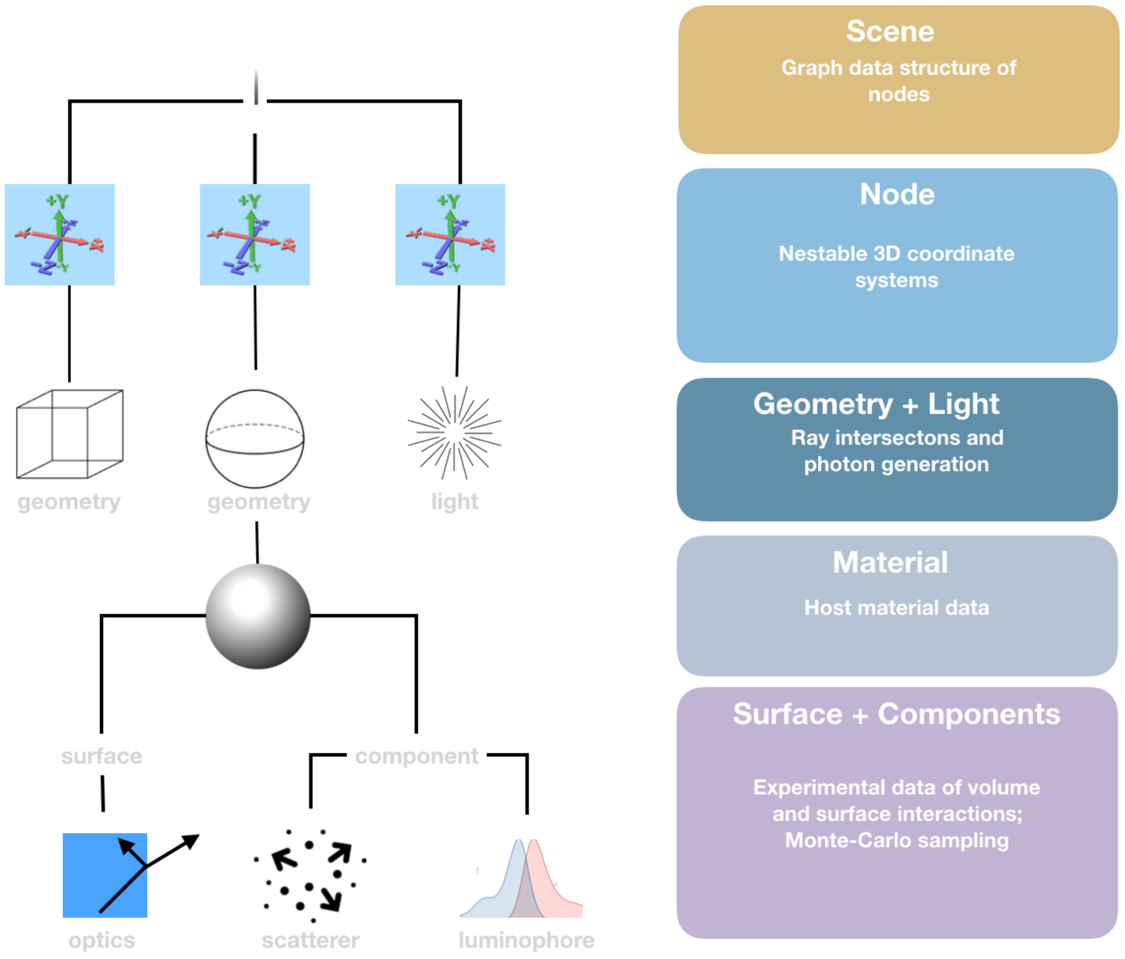

Figure S1: Code architecture of pvtrace, showing the top-down design. All objects exist within a scene, which contains nodes of geometry or light. General material properties such as refractive index can be defined for each geometry, or more specific properties such as surface characteristics, absorption/scattering, and emission can also be set. Reproduced from Farrell.[1]

S2 3D printing of Luminescent Solar Concentrators (LSCs)

LSCs were 3D printed by fused deposition modelling. The Original Prusa i3 MK3S111 kit was purchased from Prusa Research and assembled in-house according to instructions – no significant modifications were made. Ultimaker Cura with the Prusa driver installed was used for the 3D printing software. The specific printing parameters were already set when generating the STL based on the G-code and were not changed.

Before printing the LSC part, the surface was wiped with acetone wipes to eliminate any contaminants on the surface. The extruding nozzle was also cleaned between prints. The actual 3D printing of the part was completely handled by the G-code generated by Cura, with the settings set by the user. Post-printing, the part was allowed to cool for 5-10 minutes, ensuring the part solidifies completely and preventing flexure by any post-processing. The Prusa MK3S comes with a removable base, allowing easy removal of parts. The base was removed from the 3D printed and then flexed until the part detached.

Ultimaker Cura is a free commercial software used for 3D printing. Its primary purpose is converting STLs into instructions for Ultimaker 3D printers. Since the Prusa MK3S printer was used in this study, a driver was downloaded to make the output from Cura compatible with Prusa printers. The slicing software slices the STL part into several layers and generates a path the printer nozzle should follow for each layer. While there are default settings, the user can change various settings based on the specific application desired. For this study, the biggest changes made were changing the layer height and line width, increasing the infill percent to 100%, removing the walls, and reducing the print speed. The temperature had to be fine-tuned for PMMA, which is not as commonly printed as (acrylonitrile butadiene styrene) ABS or poly(lactic acid) (PLA). Fan cooling of the part was also turned off to allow fusing of layers. The infill pattern was concentric.

Line width and layer height were also optimized to maximize transparency. Theoretically, a larger line width would reduce the number of optical interfaces between a re-emitted wave and the edge of the part. A smaller layer height would make each printed path have a more suitable cross-section profile for optical transparency. The layer height was reduced to 0.05 mm and the line width set to 0.4 mm.

The Cura settings used for this study can be found in profiles available on the author's Github.[2]

S3 Experimental characterization of LSCs

An Abet Technologies Class ABB solar simulator was used as the light source for optical efficiency measurements. It was fitted with an AM1.5G filter to mimic the spectrum of actual sunlight. (Figure S2). The height of the solar simulator above the sample was first calibrated using a reference solar cell of known efficiency coupled to a Keithley 2401 sourcemeter such that the intensity of light on the sample was 1000±10 W/m². The LSC was placed on a custom-designed sample holder (Figure S3) and a single edge of the LSC was placed against to the input port to an INS125 integrating sphere (225-1400 nm, International Light Technologies), that was connected that was connected to a spectroradiometer (SpectriLight ILT 950) via a fiber optic cable. All measurements were performed on a black absorptive background.

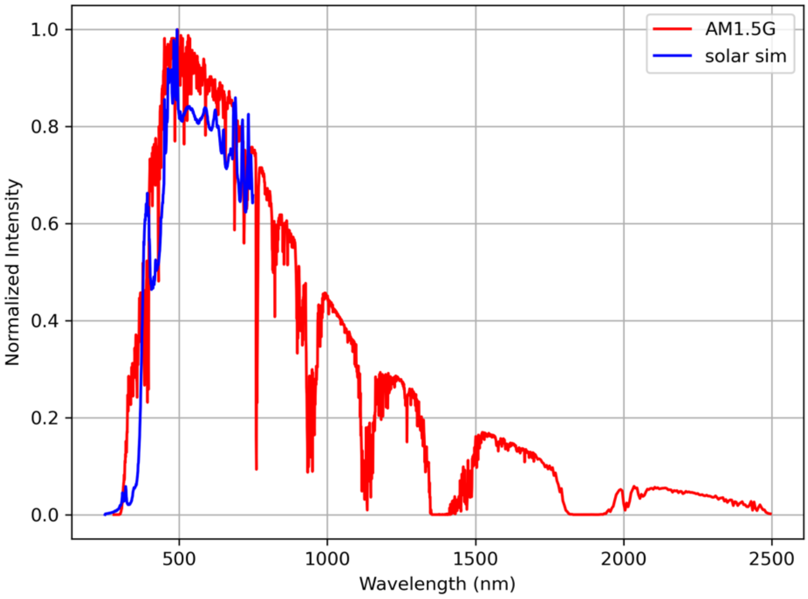

Figure S2: Comparison of AM1.5G solar spectrum provided by NREL[a] and the experimental solar simulator output (Abett Technologies Class ABB) in the spectral region of interest.

[a] Reference Air Mass 1.5 Spectra I Grid Modernization I NREL, https://www.nrel.gov/grid/solar-resource/spectra-am1.5.html, (accessed 23 July 2020).

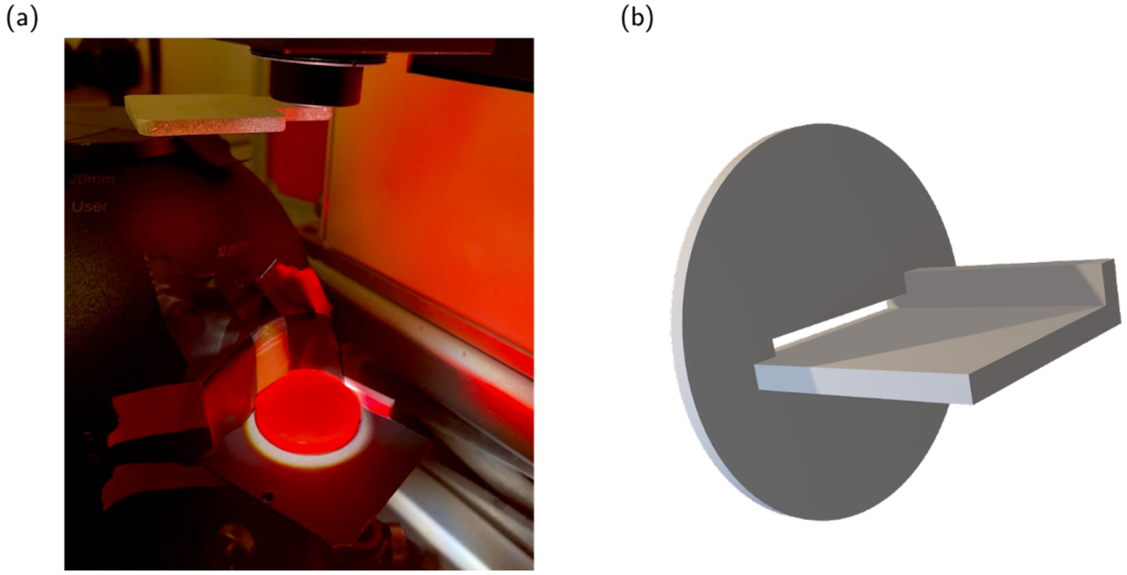

Figure S3: Experimental set-up used to determine the optical efficiency of LSCs. (a) Photograph of the experimental setup with a solar simulator shining on the top of a cut circular LSC, and the edge emitting into an integrating sphere spectrometer. (b) CAD model of the sample holder printed for the purpose of holding the LSC, serving as a mask for emitted light, and blocking out environmental light to limit noise and interference with results.

The parameters used to characterize the optical performance of LSCs are the internal photon efficiency, η_{int} and external photon efficiency, η_{ext}, defined by the following equations:[3]

$$\eta_{int} = \frac{N_{ph-out}}{N_{ph-abs}} = \frac{\sum_{i=1}^{i=n} \int_{\lambda_1}^{\lambda_2} P_{out,i}(\lambda)\frac{\lambda}{hc}d\lambda}{\int_{\lambda_1}^{\lambda_2} P_{abs}(\lambda)\frac{\lambda}{hc}d\lambda} = \frac{\sum_{i=1}^{i=n} \int_{\lambda_1}^{\lambda_2} P_{out,i}(\lambda)\frac{\lambda}{hc}d\lambda}{\int_{\lambda_1}^{\lambda_2} P_{in}(\lambda)(1-10^{-A(\lambda)})\frac{\lambda}{hc}d\lambda} \quad \text{(Eq. S1)}$$

$$\eta_{ext} = \frac{N_{ph-out}}{N_{ph-in}} = \frac{\sum_{i=1}^{i=n} \int_{\lambda_1}^{\lambda_2} P_{out,i}(\lambda)\frac{\lambda}{hc}d\lambda}{\int_{\lambda_1}^{\lambda_2} P_{in}(\lambda)\frac{\lambda}{hc}d\lambda} \quad \text{(Eq. S2)}$$

where N_{ph-out} is the total number of edge-emitted photons summed over all edges of the LSC, N_{ph-abs} is the total number of photons absorbed by the LSC, and N_{ph-in} is the total number of photons incident on the top surface of the LSC. N_{ph-out} is obtained from the sum of the output power spectra, $P_{i(out)}(\lambda)$, measured for each edge of the LSC (in W nm^{-1}), where λ is the wavelength of light (in nm). $P_{in}(\lambda)$ is the input power spectrum from the solar simulator incident on the top surface of the LSC (in W nm^{-1}), h is Planck's constant (in J s), c is the speed of light (in m s^{-1}), and $A(\lambda)$ is the absorption spectrum of the LSC. The integrations are performed over the full AM1.5G solar spectrum (250-1050 nm).

S4 Parallelization to reduce computing time

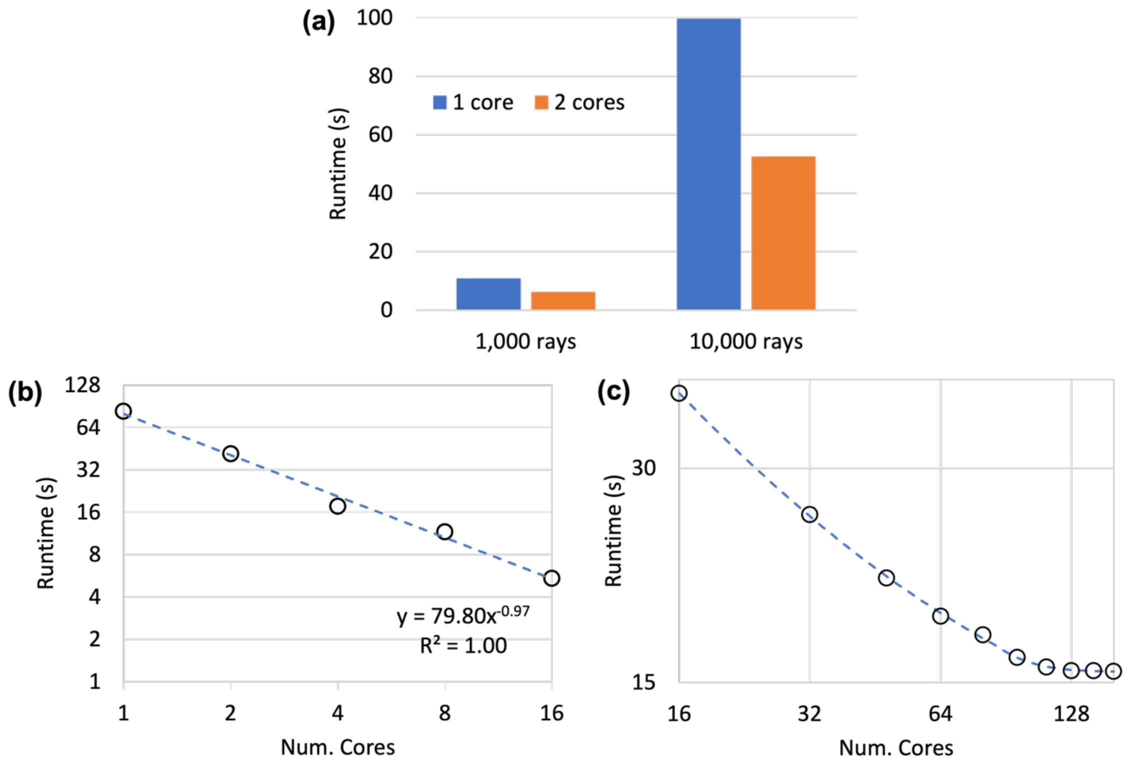

Figure S4: Effect of parallelization on simulation times for a circular LSC of 6 cm diameter and 0.32 cm thickness, using a rectangular light mask. (a) Completion time of pvtrace script for 1,000 and 10,000 rays run on a laptop, using either serial (non-parallel, 1 core) configuration or the 2 cores available. (b) Runtime for parallelized pvtrace on a single node in the CSD3 cluster,[4] for various numbers of cores. (c) Runtime for parallelized pvtrace on multiple nodes in the CSD3 cluster. Implements the Ray package for distributed computing across various nodes. Increasing cores decreases runtime to a certain extent, but overhead from various python packages increases minimum runtime.

S5 Optical properties of Lumogen Red

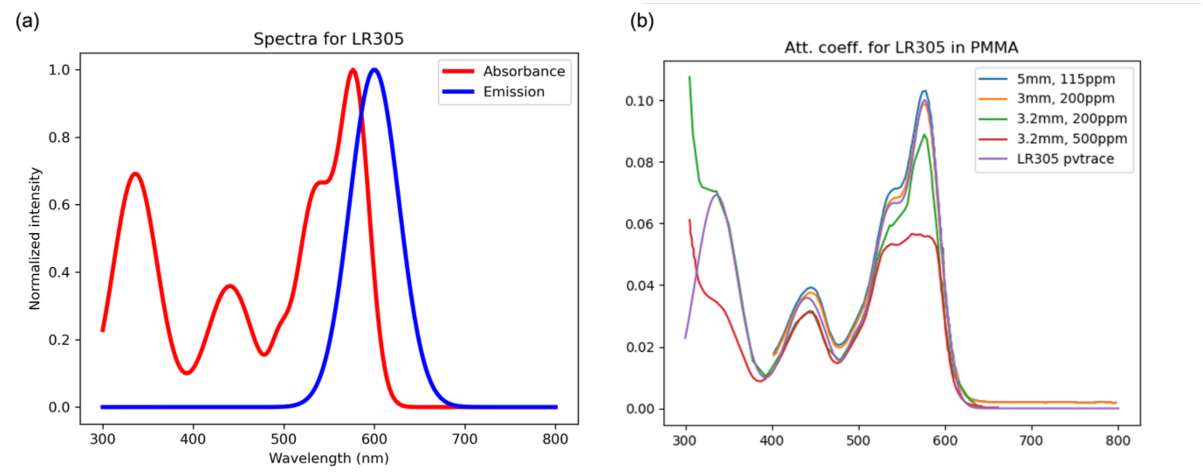

Figure S4: (a) Normalized absorbance (red) and emission (blue) spectra of Lumogen Red 305 (LR305). These spectra are in-built to pvtrace. (b) Attenuation coefficient of LR305, which has been back-calculated from absorbance for various optical path lengths and LR305 concentrations using the Beer-Lambert Law:

$$A = \epsilon c l$$

where A is the absorbance, ϵ is the attenuation coefficient, c is the concentration, and l is the optical path length. The attenuation coefficient allows calculation of absorption coefficients based on the input luminophore concentration (e.g. $\alpha = \epsilon c$ where α is the attenuation coefficient). The attenuation coefficient used in pvtrace is shown in purple, and demonstrates agreement with experimental values. Note the lower absorption at high concentrations (500 ppm) is likely due to dye aggregation effects.

S6 Input parameters and simulated outputs from pvtrace

S6.1 Case study 1: Square LSCs prepared by casting

Table S2: Input parameters used in pvtrace v2.1sv simulations (based on experimental data from the literature) for bulk square LSCs based on LR305 doped in poly(methyl methacrylate) (PMMA). We note that many of the studies did not specific the background absorption coefficients, and most of those that did, reported a constant value rather than a more realistic wavelength-dependent response.

Label	LSC Dimensions (cm)	LR305 conc. (ppm)	Refractive Index	Waveguide parasitic absorption (cm^{-1})	Light source range (nm)	Expt Light source	Emission Measurement	Ref
L1	6 x 6 x 0.32	200	1.5	0.3	300-900	AAB solar simulator	Fiber optic cable	[5]
L2	6 x 6 x 0.32	500	1.5	0.3	300-900	AAB solar simulator	Fiber optic cable	
L3	5 x 5 x 0.5	115	1.5	0.3	400-900	Solar simulator (in-house)	Solar cells (η = 16.3%)	[6]
L4	5 x 5 x 0.5	115	1.5	0.3	300-900	Outdoor sunlight	Solar cells (η = 16.3%)	
L5	5 x 5 x 0.3	49	1.5	0.02	400-700	Solar simulator	Solar cells	[7]
L6	5 x 5 x 0.3	161	1.5	0.02	400-700	Solar simulator	Solar cells	
L7	5 x 5 x 0.3	130	1.5	0.3	300-900	ABA solar simulator	Integrating sphere	[8]
L8	5 x 5 x 0.3	382	1.5	0.3	300-900	ABA Solar simulator	Integrating sphere	

Instead of importing the spectrum used in the studies (which was not available), a Planck distribution (Equation S3) at 5800K was used for the shape of the light source spectrum. Because only 10,000 – 100,000 rays are generated, a Planck distribution is sufficient to mimic solar radiation or a solar simulator. The Planck distribution is defined as:

$$B_{\lambda}(\lambda, T) = \frac{2hc^2}{\lambda^5} \frac{1}{e^{\frac{hc}{\lambda k_B T}} - 1} \quad \text{(Eq. S3)}$$

where B is the spectral radiance, λ is the wavelength, T is the temperature, h is Planck's constant, and c is the speed of light.

Table S3: Corresponding simulated (pvtrace v2.1sv) and measured external photon efficiency (η_{ext}) values, and corresponding deviation (η_{ext}) for each device.

Label	pvtrace η_{ext} (%)	Literature η_{ext} (%)	Deviation (abs)
L1	7.6	7.5	0.1
L2	8.8	8.9	0.1
L3	13.15	14.2	1.05
L4	14.71	19	4.29
L5	17.24	19.44	2.2
L6	32.18	38.64	6.46
L7	7.11	7.2	0.09
L8	10.1	8.5	1.6

S6.2 Case study 2: Unconventional LSC geometries prepared by casting

Table S4: (a) Input parameters used in pvtrace v2.1sv simulations (based on experimental data from this work) of bulk part LSCs of different geometries using the enclosing box or surface normal method of ray counting. To mimic the experimental set-up, a 1.5 mm gap between the edge of the LSC and the edge of the enclosing box was used. The LSCs were made from cast PMMA doped with LR305.

Geometry	LSC Dimensions (cm)	Top Surface Area (mm^2)	Geometric Gain, G	LR305 conc. (ppm)	Refractive Index	Waveguide parasitic absorption (cm^{-1})	Light source spectrum (nm)	Emission Measurement
Square	1.25 x 1.25 x 0.16	156	1.95	100	1.5	0.2	400-800	Integrating sphere
Circle	D = 1.4<br>H = 0.16	151	2.09	100	1.5	0.2	400-800	Integrating sphere
Hexagon	S = 1.5<br>H = 0.16	154	2.18	100	1.5	0.2	400-800	Integrating sphere
Triangle	S = 1.8<br>H = 0.16	145	1.65	100	1.5	0.2	400-800	Integrating sphere

Table S5: Corresponding simulated (pvtrace v2.1sv) and measured external photon efficiency (η_{ext}) values, and corresponding deviation (η_{ext}) for each device.

Geometry	Measured η_{ext} (%)	pvtrace η_{ext} (%) (Enclosing Box)	Measured vs. encl. box deviation (abs, %)	pvtrace η_{ext} (%) (Surface Normal)
Square	4.96	5.08	0.12	11.28
Circle	4.82	4.99	0.17	10.92
Hexagon	6.06	6.34	0.28	12.10
Triangle	4.84	4.80	0.04	12.32

Table S6: Simulated (pvtrace v2.1sv) external photon efficiency for a variety of hypothetical bulk LSC designs using enclosing box or surface normal methods of ray counting. The sample numbers correspond to the bulk LSCs illustrated in Figure 8. For all parts the following conditions were applied: (i) top surface area of 6.25 cm^2; (ii) thickness of 0.21 cm; (iii) LR305 concentration = 500 ppm; (iv) refractive index = 1.5; (v) waveguide parasitic absorption of 0.525 cm^{-1}; (vi) incident spectrum of 300-900 nm. The x- and y- dimensions of the enclosing box were set 101% of the actual x- and y-dimensions of the LSC, while the z-dimension was 110% of the actual z-dimension of the LSC.

**Sample**	**LSC Shape**	η_{ext}**(%) surf. norm.**	η_{ext}**(%) encl. box**
1	Square	10.68	10.49
2	Hexagon	11.72	7.47
3	Triangle	15.22	6.65
4	Circle (cut)	10.61	7.98
5	Circle	10.42	6.74
6	Cylinder Array	18.17	18.52
7	Cylinder Array (Hollow)	20.69	18.99
8	Leaf Roof	14.98	5.71
9	Leaf	10.42	5.03
10	Cylinder Array (vertical)	21.19	16.53

S6.3 Case study 3: 3D printed LSCs

Table S7: Input parameters used in pvtrace v2.1sv simulations (based on experimental data from this work) of 3D printed LSCs. The sample numbers correspond to the printed LSCs illustrated in Figure 11; sample preparation as described in Section 2. To mimic the experimental set-up, a 1.5 mm gap between the edge of the LSC and the edge of the enclosing box was used. The surface normal method of ray counting was also evaluated. The top surface area and geometric gain are the same as Table S4.

Geometry	LSC Dimensions (cm)	LR305 conc. (ppm)	Refractive Index	Waveguide parasitic absorption (cm^{-1})	Light source spectrum (nm)	Emission Measurement
Square	1.25 x 1.25 x 0.16	100	1.5	5	400-800	Integrating sphere
Circle	D = 1.4<br>H = 0.16	100	1.5	5	400-800	Integrating sphere
Hexagon	S = 1.5<br>H = 0.16	100	1.5	5	400-800	Integrating sphere
Triangle	S = 1.8<br>H = 0.16	100	1.5	5	400-800	Integrating sphere

Table S8: Corresponding simulated (pvtrace v2.1sv) and measured external photon efficiency (η_{ext}) values, and corresponding deviation (η_{ext}) for each device.

Geometry	Measured η_{ext} (%)	pvtrace η_{ext} (%) (Enclosing Box)	Measured vs. encl. box deviation (abs, %)	pvtrace η_{ext} (%) (Surface Normal)
Square	3.95	4.20	0.25	4.88
Circle	4.86	4.61	0.25	5.32
Hexagon	5.67	4.91	0.76	5.11
Triangle	5.45	5.65	0.20	5.58

We can also apply pvtrace v2.1.sv to extrapolate the optical performance of higher-quality parts. The 3D printed parts had much higher parasitic absorption from the waveguide, compared to the laser cut bulk parts. This was primarily due to the in-house filament preparation process, which resulted in bubbles being formed along some parts of the filament. With industrial filament preparation processes, higher-quality filament and therefore higher-quality parts could be fabricated. Reducing the waveguide parasitic absorption term in pvtrace shows the potential performance of 3D printed parts. Further, switching to the *surface normal* method provides an indication of the potential performance with an optimized measurement technique. The results of this extrapolation are shown in Table S9.

Table S9: Simulated LSC efficiency for square devices with various absorption coefficients. (high abs) column corresponds to the absorption coefficients in the current study (~5 cm^{-1}), (med abs) corresponds to 66% of the current absorption coefficient, and (low abs) corresponds to absorption matching the commercial PMMA+LR305 slab (0.2 cm^{-1}). All other parameters are provided in Table S7.

Geometry	pvtrace surf. norm. η_{ext} (high abs, %)	pvtrace surf. norm. η_{ext} (medium abs, %)	pvtrace surf. norm. η_{ext} (low abs, %)
Square, bulk	–	–	11.28
Square, 3DP	4.88	7.31	15.97

Table S10: Simulated (pvtrace v2.1sv) external photon efficiency for a variety of hypothetical 3D printed LSC designs using enclosing box or surface normal methods of ray counting. The sample numbers correspond to the 3D printed LSCs illustrated in Figure 11. For all parts the following conditions were applied: (i) top surface area of 6.25 cm^2; (ii) thickness of 0.21 cm; (iii) LR305 concentration = 500 ppm; (iv) refractive index = 1.5; (v) waveguide parasitic absorption of 0.525 cm^{-1}; (vi) incident spectrum of 300-900 nm. The x- and y- dimensions of the enclosing box were set 101% of the actual x- and y- dimensions of the LSC, while the z-dimension was 110% of the actual z-dimension of the LSC.

Sample	LSC Shape	η_{ext}(%) surf. norm.	η_{ext}(%) encl. box
(11)	Square	18.76	19.28
(12)	Square, vert	2.93	9.63
(13)	Hexagon	13.93	11.76
(14)	Triangle	19.48	11.47
(15)	Circle, cut	15.67	11.52
(16)	Circle	13.76	12.28
(17)	Cylinder Array, vert	4.68	15.05
(18)	Cylinder Array, hollow, vert	4.91	14.06
(19)	Leaf Roof	23.29	15.69
(20)	Leaf	13.98	10.11
(21)	Cylinder Array	15.77	12.20

S7 Measured versus simulated optical efficiencies

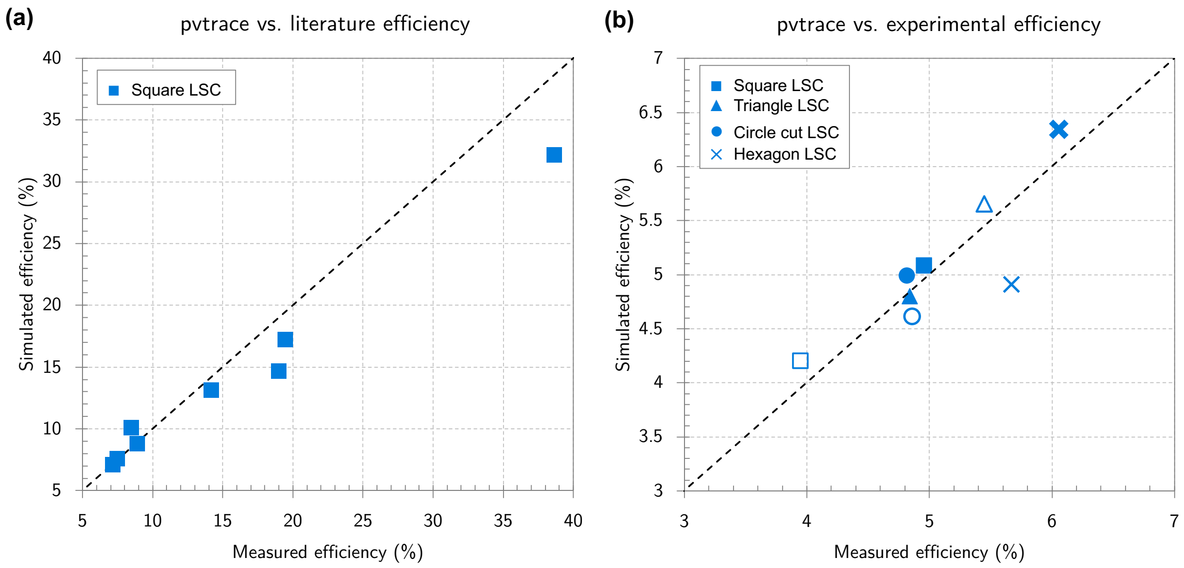

Figure S5: Comparison of measured vs. simulated (pvtrace v2.1.sv) results for LSC optical efficiency. (a) Literature data for bulk square LSCs and (b) LSCs fabricated for this study of various geometries and manufacturing techniques. Marker shapes reflect LSC geometry, with X representing the hexagon. Filled markers are bulk LSCs, while hollow ones are 3D printed LSCs. Simulated results use the enclosing box method.

S8 References

(1) Farrell D. J., pvtrace: Optical ray tracing for luminescent materials and spectral converter photovoltaic devices, https://github.com/danieljfarrell/pvtrace (Accessed: 2021-02-19)

(2) *shomikverma/CustomCuraProfiles*. https://github.com/shomikverma/CustomCuraProfiles (accessed 2022-10-24).

(3) Debije, M. G.; Evans, R. C.; Griffini, G. Laboratory Protocols for Measuring and Reporting the Performance of Luminescent Solar Concentrators. *Energy Environ. Sci.* **2021**, *14* (1), 293–301. https://doi.org/10.1039/D0EE02967J.

(4) Cambridge Service for Data-Driven Discovery (CSD3) | Research Computing Services.

(5) Zettl, M.; Mayer, O.; Klampaftis, E.; Richards, B. S. Investigation of Host Polymers for Luminescent Solar Concentrators. *Energy Technol.* **2017**, *5* (7), 1037–1044. https://doi.org/10.1002/ente.201600498.

(6) Desmet, L.; Ras, A. J. M.; de Boer, D. K. G.; Debije, M. G. Monocrystalline Silicon Photovoltaic Luminescent Solar Concentrator with 4.2% Power Conversion Efficiency. *Opt. Lett.* **2012**, *37* (15), 3087–3087. https://doi.org/10.1364/ol.37.003087.

(7) Bose, R.; Farrell, D. J.; Chatten, A. J.; Pravettoni, M.; Buchtemann, A.; Barnham, K. W. J. Novel Configurations of Luminescent Solar Concentrators. *Compil. State---Art PV Sol. Technol. Deploy.* **2007**, 210–214.

(8) Debije, M. G.; Teunissen, J. P.; Kastelijn, M. J.; Verbunt, P. P. C.; Bastiaansen, C. W. M. The Effect of a Scattering Layer on the Edge Output of a Luminescent Solar Concentrator. *Sol. Energy Mater. Sol. Cells* **2009**, *93* (8), 1345–1350. https://doi.org/10.1016/j.solmat.2009.02.013.
